# Integrated assessment of non-invasive diagnostic tools for bladder cancer: a network meta-analysis

**DOI:** 10.3389/fonc.2025.1649420

**Published:** 2025-10-17

**Authors:** Desheng Zhang, Bo Chen, Junjiang Ye, Yunjin Bai, Ping Han

**Affiliations:** ^1^ Department of Urology, West China Hospital, Sichuan University, Chengdu, Sichuan, China; ^2^ Institute of Urology, West China Hospital, Sichuan University, Chengdu, Sichuan, China

**Keywords:** bladder cancer, non-invasive diagnosis, biomarker, network meta-analysis, superiority index

## Abstract

**Background:**

Bladder cancer is a common and recurrent urologic malignancy. Although cystoscopy remains the diagnostic gold standard, its invasiveness, high cost, and limited patient compliance restrict its routine application. Non-invasive biomarkers have emerged as promising alternatives; however, their diagnostic performance has not yet been systematically compared across studies.

**Methods:**

We searched PubMed, Embase, Web of Science, and the Cochrane Library for publications available until February 21, 2025. A total of 26 original studies on non-invasive diagnostic methods for bladder cancer were included. A Bayesian network meta-analysis was performed to compare biomarkers derived from urine and blood samples. Diagnostic performance was evaluated using sensitivity, specificity, diagnostic odds ratio (DOR), and the superiority index. Subgroup analyses were conducted for microRNAs and combined biomarker strategies. Study quality was assessed with the QUADAS-2 tool, and model convergence was verified using Rhat values.

**Results:**

Significant differences in biomarker performance were identified. In urine samples, angiogenin achieved the highest superiority index (5.28), while miR-125b was the best-performing microRNA (10.97). Combined detection strategies involving *TERT/FGFR3/TP53/PIK3CA/KRAS* demonstrated strong performance (8.54). In blood samples, miR-181b-5p and fibronectin had an index of 3.02, whereas miR-301a-3p exhibited the greatest superiority (50.71).

**Conclusion:**

This study is the first to systematically compare non-invasive bladder cancer biomarkers within a Bayesian framework. Specific microRNAs and combined detection strategies demonstrated robust diagnostic potential, providing a promising alternative to cystoscopy, particularly for early screening and patient monitoring.

**Systematic review registration:**

https://www.crd.york.ac.uk/PROSPERO/, identifier CRD420251018161.

## 1 Introduction

Bladder cancer poses a serious global challenge and is one of the leading tumors affecting the urinary tract. In the year 2020, roughly 573,000 individuals were newly affected worldwide, and around 213,000 deaths were attributed to this disease, highlighting its heavy impact on population health (1). The Cancer Statistics 2024 report further predicts that in 2025, the United States will face approximately 84,870 new diagnoses and 17,420 related deaths (2). Pathologically, bladder cancer encompasses several subtypes, including urothelial carcinoma, squamous cell carcinoma, and adenocarcinoma, with urothelial carcinoma accounting for more than 90% of cases (3). Clinically, painless hematuria represents the most common manifestation. Many patients present with asymptomatic microscopic hematuria, which often leads to delayed diagnosis (4). Importantly, bladder cancer is marked by frequent relapse and requires continuous monitoring, resulting in exceptionally high per-patient management costs.

In urological practice, cystoscopy and bladder biopsy are routinely employed for the diagnosis of bladder cancer (5). However, current diagnostic methods have several important limitations. Although conventional cystoscopy remains the diagnostic gold standard, its invasive nature can cause patient discomfort and potential complications. In addition, advanced cystoscopy equipment is costly and associated with a relatively high false-positive rate. Urine cytology, while non-invasive and easy to perform, demonstrates low sensitivity for low-grade bladder cancer, leading to frequent missed diagnoses. Furthermore, its accuracy depends heavily on the expertise of pathologists, thereby introducing substantial subjectivity (5). Consequently, the identification of effective and non-invasive diagnostic markers for early-stage bladder cancer remains an urgent research priority.

In recent years, liquid biopsy technologies have attracted growing attention because of their considerable potential for non-invasive tumor detection. Research on urinary biomarkers has advanced rapidly, providing novel approaches for the early detection, diagnosis, and monitoring of urological malignancies such as bladder cancer. Among urinary biomarkers, emerging candidates based on cfDNA, non-coding RNAs, proteins, extracellular vesicles (EVs), and metabolites have shown promising potential in bladder cancer diagnosis without invasive procedures (6). Previous meta-analyses have primarily employed pairwise comparisons, which are limited to intra-category evaluations of single biomarker types. However, these studies lack cross-category comparisons and systematic assessments of combined diagnostic markers or subgroup performance, thereby hindering a thorough grasp of the complementary roles of different biomarker combinations in bladder cancer diagnosis. To address these limitations, the present study employs a network meta-analysis to systematically integrate original research data on non-invasive diagnostic tools for bladder cancer. The objective is to provide a comprehensive evaluation of the diagnostic performance of biomarkers derived from urine and blood, bridge existing research gaps, and establish a scientific foundation for clinical practice.

## 2 Methods

This study was conducted in strict accordance with the extended PRISMA guidelines, which provide standardized reporting criteria specifically designed for network meta-analyses (7). The study protocol was registered with PROSPERO (International Prospective Register of Systematic Reviews) under registration number CRD420251018161. Further details are available at https://www.crd.york.ac.uk/PROSPERO/.

### 2.1 Retrieval of literature and study selection

Databases including PubMed, Embase, Web of Science, and the Cochrane Library were screened from their inception up to February 21, 2025 (last update) to identify studies evaluating the diagnostic accuracy of non-invasive tools for bladder cancer. The search strategy combined MeSH terms, relevant keywords, and free-text vocabulary to maximize retrieval sensitivity. In addition, the reference sections of all selected studies were manually reviewed to ensure that no potentially relevant publications were overlooked. The core elements of this study were defined according to the PICOS framework: population (P)—human patients with confirmed or suspected bladder cancer; intervention (I)—diagnostic evaluation using non-invasive biomarkers, including microRNAs, protein markers, or multi-gene panels; comparison (C)—conventional diagnostic methods (such as cytology and cystoscopy) or other biomarkers; outcomes (O)—diagnostic accuracy measures, including sensitivity, specificity, and area under the curve (AUC), or studies providing sufficient data to construct 2 × 2 contingency tables; and study design (S)—original prospective or retrospective diagnostic accuracy studies.

The detailed search strategy was as follows: (bladder cancer OR bladder neoplasm) AND (cell-free nucleic acid OR survivin OR microRNAs OR angiogenin OR cytology OR nuclear matrix protein 22 OR fibronectin OR UCA1) (**Supplementary Table S1**). We also made every effort to obtain the full texts of all potentially eligible studies through database searches, institutional access, and direct contact when necessary. Based on the information above, the inclusion and exclusion criteria were further refined. The inclusion criteria were as follows: (1) original research articles, (2) evaluation of the diagnostic performance of one or more non-invasive diagnostic tools, (3) studies that reported sensitivity and specificity or provided sufficient information (e.g., ROC curves) to calculate these metrics and construct 2 × 2 tables, (4) published within the past 5 years, (5) written in English, and (6) full text is available.

The exclusion criteria are as follows: (1) systematic reviews and meta-analyses, (2) non-original research types such as case reports, letters, guidelines, conference abstracts, editorials, notes, surveys, retraction statements, and preprints, (3) studies unrelated to the research topic, (4) animal studies, (5) non-English publications, (6) studies without accessible full text, and (7) studies that did not report diagnostic metrics and from which 2 × 2 table data could not be extracted.

### 2.2 Data extraction and quality assessments

All included references were imported into EndNote (version 20), and duplicate records were removed. Two researchers (ZDS and CB) independently screened the titles and abstracts according to the predefined inclusion criteria. The full texts of potentially relevant studies were then reviewed to determine final eligibility. To ensure the accuracy and consistency of the extracted data, both researchers performed independent data extraction for all of the included studies. Discrepancies were settled by discussing with a third researcher (YJJ). For studies meeting the inclusion criteria, the data extracted included article title, lead author, year of publication, study design, country where the research was conducted, number of participants in patient and control groups, age and sex distribution (female/male), type and source of the evaluated biomarkers, and key diagnostic metrics such as area under the curve (AUC), sensitivity, and specificity. To systematically assess study quality and potential bias in the included diagnostics, the revised QUADAS-2 tool (Quality Assessment of Diagnostic Accuracy Studies) was applied (8). This framework assesses studies across four domains: participant selection, use of the index assessment, choice of comparator standard, and appropriateness of timing and sequencing. Every domain was critically examined to identify potential sources of bias and to ensure that the overall body of evidence maintained scientific rigor and credibility (8).

### 2.3 Statistical analysis

All computations were performed with R software (version 4.1.2). For studies lacking explicit sensitivity and specificity values, ROC curves were digitized with Origin (2025) software to extract the corresponding metrics. A random-effects model was applied to synthesize key diagnostic performance measures, including sensitivity, specificity, DOR, superiority index, comparative sensitivity, and comparative specificity, with corresponding 95% confidence intervals (CIs). To systematically compare the effectiveness of non-invasive diagnostic methods in detecting bladder malignancy, a Bayesian-based network meta-analysis was performed. Compared with traditional frequentist methods, the Bayesian framework is better suited for handling complex models, as it allows the inclusion of study-specific covariates and provides stable and accurate estimates even with limited data. Moreover, it enables probabilistic inference and facilitates the ranking of clinical effectiveness (9).

The original studies included in this analysis evaluated a range of non-invasive diagnostic methods, such as cytology, nucleic acid biomarkers in urine (e.g., microRNAs, UCA1, cell-free nucleic acids), and protein biomarkers (e.g., NMP22, survivin, fibronectin, angiogenin). A two-way ANOVA model was applied to calculate the posterior estimates for each method, and 95% credible intervals were reported for the six diagnostic metrics described above (10). The analysis was conducted using non-informative priors and was based on two Markov chains. A burn-in period of 1,000 iterations was applied to ensure model stability, followed by 10,000 simulation iterations for final estimation (11). Model convergence was assessed by evaluating the Rhat values, thereby confirming the reliability of the posterior distributions (12). The superiority index was also calculated to quantify relative performance across diagnostic tools. A superiority index approaching ∞ indicates superior diagnostic performance, values near 0 suggest poor performance, and values close to 1 reflect comparable effectiveness (13). Diagnostic effectiveness was further assessed using multiple indicators, including sensitivity, discriminative power measured by the DOR, comparative sensitivity and specificity, and a performance-ranking index. Additionally, Rhat values were used to verify convergence and stability of the Markov Chain Monte Carlo (MCMC) simulations, thereby strengthening the reliability of the analytical results.

## 3 Results

### 3.1 Literature identification and study features

**Figure 1:**
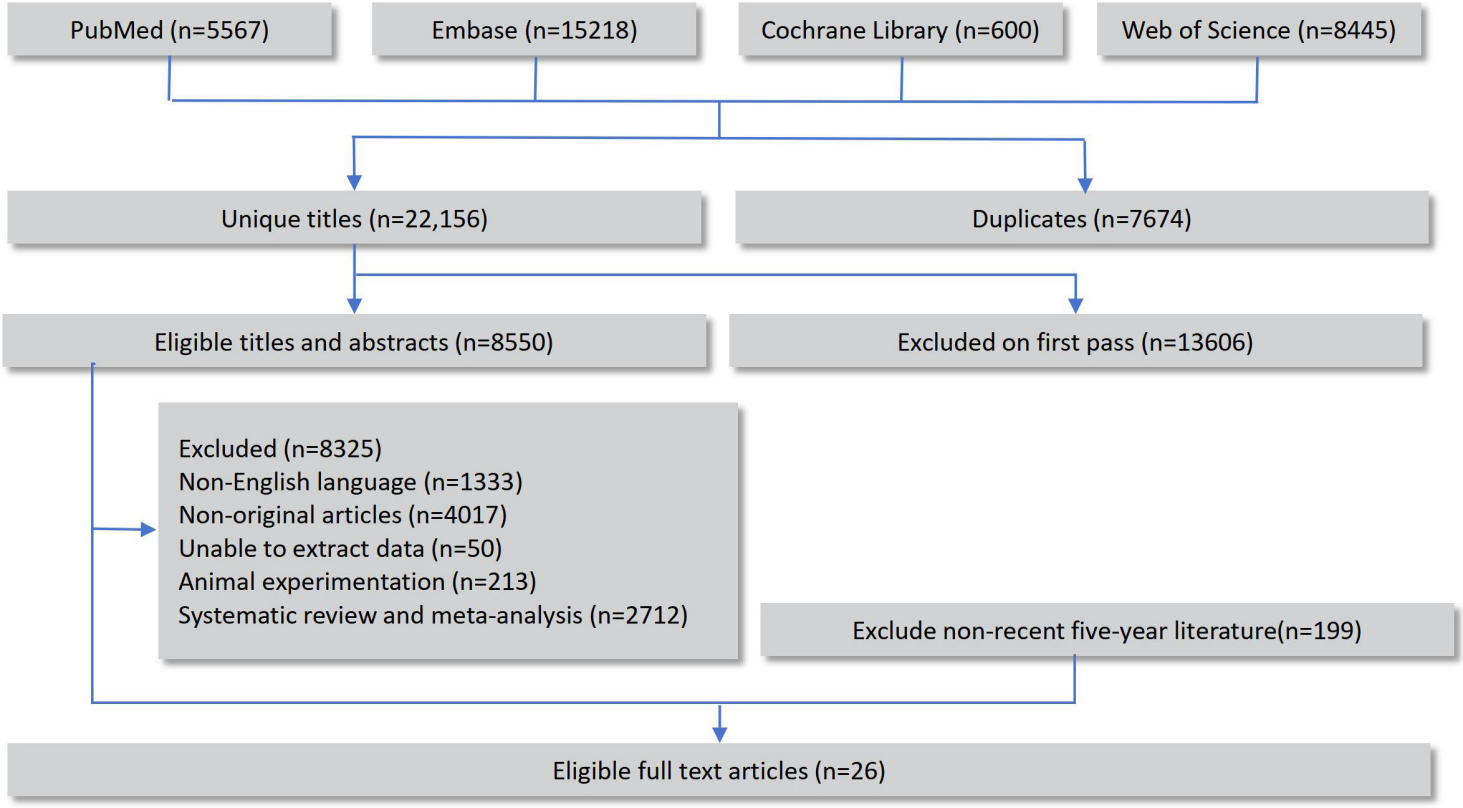
Flowchart of the screening process for non-invasive diagnostic methods of bladder cancer.

A total of 29,830 records were retrieved through database searches, including 8,445 from Web of Science, 5,567 from PubMed, 15,218 from Embase, and 600 from the Cochrane Library. After removing duplicates, the remaining records underwent preliminary screening and full-text review, and 26 original studies that fulfilled the eligibility criteria were ultimately incorporated into the network meta-analysis (14–39). The overall selection workflow is illustrated in **Figure 1**.

Key features of the eligible studies are outlined in **Table 1**. Two types of biomarker sources were analyzed: urine and blood. A total of seven types of urine-derived biomarkers were analyzed, including angiogenin, microRNAs, *cell-free DNA* (*TERT*), survivin, UCA1, urine cytology, and NMP22 (**Table 2**). Among the urine-based biomarkers, subgroup analyses were performed for microRNAs and combined detection strategies. In total, 20 different microRNAs and 15 biomarker combinations were evaluated for comparative diagnostic performance (**Table 3**). The blood-derived biomarkers encompassed five categories, namely: miR-181b-5p/miR-183-5p/miR-199-5p/miR-211-3p, fibronectin, miR-132-3p/miR-7-5p/miR-148b-3p, angiogenin, and other microRNAs (**Table 4**). The blood-based subgroup analysis included diagnostic performance data for 32 different microRNAs (**Table 5**).

**Table 1:** Study characteristics.

Study	Type	Country	Patients			Control			Biomarker	Sources	AUC	Sensitivity	Specificity	Tp	Fp	Fn	Tn
			*n*	Age	Gender (F/M)	*n*	Age	Gender (F/M)									
Erdmann 2020 (13)	CCS	Germany	104	NA	21/83	46	NA	16/30	miR-21	Urine	0.581	0.865	0.304	90	32	14	14
Erdmann 2020 (13)	CCS	Germany	104	NA	21/83	46	NA	16/30	miR-96	Urine	0.605	0.298	0.913	31	4	73	42
Erdmann 2020 (13)	CCS	Germany	104	NA	21/83	46	NA	16/30	miR-125b	Urine	0.714	0.885	0.543	92	21	12	25
Erdmann 2020 (13)	CCS	Germany	104	NA	21/83	46	NA	16/30	miR-126	Urine	0.667	0.885	0.217	92	36	12	10
Erdmann 2020 (13)	CCS	Germany	104	NA	21/83	46	NA	16/30	miR-145	Urine	0.687	0.5	0.848	52	7	52	39
Erdmann 2020 (13)	CCS	Germany	104	NA	21/83	46	NA	16/30	miR-183	Urine	0.72	0.817	0.609	85	18	19	28
Erdmann 2020 (13)	CCS	Germany	104	NA	21/83	46	NA	16/30	miR-205	Urine	0.537	0.779	0.435	81	26	23	20
Erdmann 2020 (13)	CCS	Germany	104	NA	21/83	46	NA	16/30	miR-210	Urine	0.526	0.663	0.5	69	23	35	23
Erdmann 2020 (13)	CCS	Germany	104	NA	21/83	46	NA	16/30	miR-221	Urine	0.772	0.779	0.674	81	15	23	31
Erdmann 2020 (13)	CCS	Germany	104	NA	21/83	46	NA	16/30	VUC	Urine	NA	0.769	1	80	0	24	46
Erdmann 2020 (13)	CCS	Germany	104	NA	21/83	46	NA	16/30	6 miRs-96/125b/126/145/183/221	Urine	NA	0.731	0.935	76	3	28	43
Erdmann 2020 (13)	CCS	Germany	104	NA	21/83	46	NA	16/30	4 miRs-125b/145/183/221	Urine	NA	0.731	0.957	76	2	28	44
Erdmann 2020 (13)	CCS	Germany	104	NA	21/83	46	NA	16/30	6 miRs-96/125b/126/145/183/221 + VUC	Urine	NA	0.808	0.935	84	3	20	43
Erdmann 2020 (13)	CCS	Germany	104	NA	21/83	46	NA	16/30	4 miRs-125b/145/183/221 + VUC	Urine	NA	0.846	0.957	88	2	16	44
Ou 2020 (14)	Cohort study	China	92	63 (15–89)	14/78	33	54 (21–81)	13/20	*TERT*	Urine	NA	0.46	1	42	0	50	33
Ou 2020 (14)	Cohort study	China	92	63 (15–89)	14/78	33	54 (21–81)	13/20	*TERT/FGFR3/TP53/PIK3CA/KRAS*	Urine	0.9401	0.87625	0.95035	81	2	11	31
Ou 2020 (14)	Cohort study	China	92	63 (15–89)	14/78	33	54 (21–81)	13/20	*TERT*	Urine	NA	0.48	1	44	0	48	33
Ou 2020 (14)	Cohort study	China	92	63 (15–89)	14/78	33	54 (21–81)	13/20	*TERT/FGFR3/TP53/HRAS/PIK3CA/KRAS/ERBB2* (urine sediments)	Urine	0.9071	0.86106	0.88581	79	4	13	29
Szyman´ska 2020 (15)	CCS	Poland	60	41–88 (66)	10/50	28	50–81 (67)	5/23	ANG	Urine	0.91	0.833	0.929	50	2	10	26
Shal 2021 (16)	CCS	Egypt	51	59.5 ± 3.2	9/42	49	58.23 ± 3.057	8/41	miR-96	Urine	0.85	0.804	0.918	41	4	10	45
Shal 2021 (16)	CCS	Egypt	51	59.5 ± 3.2	9/42	49	58.23 ± 3.057	8/41	miR-183	Urine	0.83	0.784	0.816	40	9	11	40
Shal 2021 (16)	CCS	Egypt	51	59.5 ± 3.2	9/42	49	58.23 ± 3.057	8/41	Cytology	Urine	0.69	0.373	1	19	0	32	49
Shal 2021 (16)	CCS	Egypt	51	59.5 ± 3.2	9/42	49	58.23 ± 3.057	8/41	Combined miR-96 and miR-183	Urine	0.88	0.882	0.878	45	6	6	43
Shal 2021 (16)	CCS	Egypt	51	59.5 ± 3.2	9/42	49	58.23 ± 3.057	8/41	Combined miR-96 and cytology	Urine	0.87	0.824	0.918	42	4	9	45
Shal 2021 (16)	CCS	Egypt	51	59.5 ± 3.2	9/42	49	58.23 ± 3.057	8/41	Combined miR-183 and cytology	Urine	0.85	0.804	0.918	41	4	10	45
Gong 2021 (17)	CCS	China	61	70 (60–76)	18/43	52	NA	17/35	Cytology	Urine	0.59	0.18	1	11	0	50	52
Gong 2021 (17)	CCS	China	61	70 (60–76)	18/43	52	NA	17/35	NMP22	Urine	0.69	0.59	0.79	36	11	25	41
Gong 2021 (17)	CCS	China	61	70 (60–76)	18/43	52	NA	17/35	Survivin	Urine	0.84	0.75	0.83	46	9	15	43
Gong 2021 (17)	CCS	China	61	70 (60–76)	18/43	52	NA	17/35	NMP22 + cytology	Urine	0.72	0.61	0.79	37	11	24	41
Gong 2021 (17)	CCS	China	61	70 (60–76)	18/43	52	NA	17/35	Survivin + cytology	Urine	0.81	0.77	0.83	47	9	14	43
Gong 2021 (17)	CCS	China	61	70 (60–76)	18/43	52	NA	17/35	Survivin + NMP22 + cytology	Urine	0.95	0.77	0.83	47	9	14	43
Lin 2021 (18)	CCS	China	53	65 (52–69)	13/40	62	62 (54–68)	16/35	miR-93-5p	Urine	0.838	0.741	0.902	39	6	14	56
Lin 2021 (18)	CCS	China	53	65 (52–69)	13/40	62	62 (54–68)	16/35	miR-516a-5p	Urine	0.79	0.729	0.899	39	6	14	56
Lin 2021 (18)	CCS	China	53	65 (52–69)	13/40	62	62 (54–68)	16/35	miR-93-5p + miR-516a-5p	Urine	0.867	0.852	0.824	45	11	8	51
Lin 2021 (18)	CCS	China	53	65 (52–69)	13/40	62	62 (54–68)	16/35	Urine cytology	Urine	0.63	0.259	1	14	0	39	62
Sun 2021 (19)	CCS	China	119	65 (46–84)	44/75	60	62 (41–88)	24/36	NMP22	Urine	0.705	0.642	0.662	76	20	43	40
Szymanska 2021 (20)	CCS	Poland	60	41–88 (66)	10/50	28	50–81 (67)	5/23	ANG	Serum	0.87	0.58	0.99	35	0	25	28
Yang 2021 (21)	CCS	China	88	NA	22/66	36	NA	NA	miR-10a-5p	Plasma	0.815	0.795	0.656	70	12	18	24
Yang 2021 (21)	CCS	China	56	NA	NA	32	NA	NA	miR-10a-5p	Plasma	0.785	0.75	0.642	42	11	14	21
Bian 2022 (22)	CCS	China	50	65.14 ± 13.94	7/43	50	66.54 ± 17.16	NA	UCA1	Urine	0.813	0.71547	0.69712	36	15	14	35
Bian 2022 (22)	CCS	China	50	65.14 ± 13.94	7/43	50	66.54 ± 17.16	NA	NMP 22	Urine	0.65	0.42	0.88	21	6	29	44
Chen 2022 (23)	CCS	China	128	NA	30/98	94	NA	NA	Urine cytology	Urine	NA	0.4375	0.98956	56	1	72	93
Li 2022 (24)	CCS	China	82	NA	NA	80	NA	NA	hsa-miR-1-3p	Serum	0.682	0.7842	0.5316	64	37	18	43
Li 2022 (24)	CCS	China	82	NA	NA	80	NA	NA	hsa-miR-23b-3p	Serum	0.748	0.73484	0.59777	60	32	22	48
Li 2022 (24)	CCS	China	82	NA	NA	80	NA	NA	hsa-miR-34a-5p	Serum	0.804	0.70866	0.65893	58	27	24	53
Li 2022 (24)	CCS	China	82	NA	NA	80	NA	NA	hsa-miR-124-3p	Serum	0.688	0.58764	0.73428	48	21	34	59
Li 2022 (24)	CCS	China	82	NA	NA	80	NA	NA	hsa-miR-182-5p	Serum	0.867	0.70432	0.84148	58	13	24	67
Li 2022 (24)	CCS	China	82	NA	NA	80	NA	NA	hsa-miR-196a-5p	Serum	0.731	0.62874	0.69204	52	25	30	55
Li 2022 (25)	CCS	China	82	61.2 ± 13.5	40/42	82	62.7 ± 9.1	38/44	miR-106a-5p	Serum	0.634	0.7317	0.5366	60	38	22	44
Li 2022 (25)	CCS	China	82	61.2 ± 13.5	40/42	82	62.7 ± 9.1	38/44	miR-145-5p	Serum	0.677	0.7561	0.561	62	36	20	46
Li 2022 (25)	CCS	China	82	61.2 ± 13.5	40/42	82	62.7 ± 9.1	38/44	miR-132-3p	Serum	0.781	0.6829	0.8171	56	15	26	67
Li 2022 (25)	CCS	China	82	61.2 ± 13.5	40/42	82	62.7 ± 9.1	38/44	miR-7-5p	Serum	0.778	0.5976	0.8415	49	13	33	69
Li 2022 (25)	CCS	China	82	61.2 ± 13.5	40/42	82	62.7 ± 9.1	38/44	miR-148b-3p	Serum	0.837	0.8171	0.7195	67	23	15	59
Li 2022 (25)	CCS	China	82	61.2 ± 13.5	40/42	82	62.7 ± 9.1	38/44	The three-miRNA panel (miR-132-3p, miR-7-5p, and miR-148b-3p)	Serum	0.922	0.9024	0.8171	74	15	8	67
Li 2022 (26)	CCS	China	84	60.85 ± 1.46	20/64	84	63.06 ± 0.78	21/63	let-7c-5p	Serum	0.624	0.56756	0.68747	48	26	36	58
Li 2022 (26)	CCS	China	84	60.85 ± 1.46	20/64	84	63.06 ± 0.78	21/63	miR-9-5p	Serum	0.666	0.68	0.72	57	24	27	60
Li 2022 (26)	CCS	China	84	60.85 ± 1.46	20/64	84	63.06 ± 0.78	21/63	miR-181b-5p	Serum	0.723	0.63099	0.77285	53	19	31	65
Li 2022 (26)	CCS	China	84	60.85 ± 1.46	20/64	84	63.06 ± 0.78	21/63	miR-183-5p	Serum	0.751	0.66829	0.72852	56	23	28	61
Li 2022 (26)	CCS	China	84	60.85 ± 1.46	20/64	84	63.06 ± 0.78	21/63	miR-199a-5p	Serum	0.703	0.73346	0.58027	62	35	22	49
Li 2022 (26)	CCS	China	84	60.85 ± 1.46	20/64	84	63.06 ± 0.78	21/63	miR-221-3p	Serum	0.663	0.54527	0.66381	46	28	38	56
Li 2022 (26)	CCS	China	28	64.89 ± 2.50	5/23	28	62.50 ± 1.18	9/19	miR-181b-5p/miR-183-5p/miR-199-5p/miR-211-3p	Serum	0.925	0.8214	0.9286	23	2	5	26
Pakmanesh 2022 (27)	CCS	Iran	31	64.9 ± 9.4	5/26	50	59.6 ± 13.4	16/34	Urine cytology	Urine	NA	0.677	0.62	21	19	10	31
Qiu 2022 (28)	CCS		22	NA	NA	20	NA	NA	UCA1	Urine	0.739	0.682	0.85	15	3	7	17
Singh 2022 (29)	CCS	India	25	59 ± 8	4/21	35	52.14 ± 9.594	5/30	miR-9	Serum	0.8176	0.84	0.68	21	11	4	24
Singh 2022 (29)	CCS	India	25	59 ± 8	4/21	10	60 ± 9	0/10	miR-9	Serum	0.716	0.68	0.7	17	3	8	7
Singh 2022 (29)	CCS	India	25	59 ± 8	4/21	35	52.14 ± 9.594	5/30	miR-34a	Serum	0.7008	0.68	0.72	17	10	8	25
Singh 2022 (29)	CCS	India	25	59 ± 8	4/21	10	60 ± 9	0/10	miR-34a	Serum	0.86	0.8	0.7	20	3	5	7
Singh 2022 (29)	CCS	India	25	59 ± 8	4/21	35	52.14 ± 9.594	5/30	miR-203	Serum	0.7288	0.72	0.72	18	10	7	25
Singh 2022 (29)	CCS	India	25	59 ± 8	4/21	10	60 ± 9	0/10	miR-203	Serum	0.852	0.8	0.7	20	3	5	7
Singh 2022 (29)	CCS	India	25	59 ± 8	4/21	35	52.14 ± 9.594	5/30	miR-9	Urine	0.8978	1	0.7333	25	9	0	26
Singh 2022 (29)	CCS	India	25	59 ± 8	4/21	10	60 ± 9	0/10	miR-9	Urine	0.7867	0.8	0.6	20	4	5	6
Singh 2022 (29)	CCS	India	25	59 ± 8	4/21	35	52.14 ± 9.594	5/30	miR-34a	Urine	0.5022	0.6667	0.4667	17	19	8	16
Singh 2022 (29)	CCS	India	25	59 ± 8	4/21	10	60 ± 9	0/10	miR-34a	Urine	0.84	0.8	0.8	20	2	5	8
Singh 2022 (29)	CCS	India	25	59 ± 8	4/21	35	52.14 ± 9.594	5/30	miR-203	Urine	0.9289	0.9333	0.8	23	7	2	28
Singh 2022 (29)	CCS	India	25	59 ± 8	4/21	10	60 ± 9	0/10	miR-203	Urine	0.9667	0.9333	0.9	23	1	2	9
Wen 2022 (30)	CCS	China	80	63.3 ± 12.9	15/65	80	61.5 ± 10.3	19/61	miR-1-3p	Blood	0.651	0.70776	0.59256	57	33	23	47
Wen 2022 (30)	CCS	China	80	63.3 ± 12.9	15/65	80	61.5 ± 10.3	19/61	miR-30a-5p	Blood	0.692	0.72949	0.57173	58	34	22	46
Wen 2022 (30)	CCS	China	80	63.3 ± 12.9	15/65	80	61.5 ± 10.3	19/61	miR-100-5p	Blood	0.738	0.72583	0.68844	58	25	22	55
Wen 2022 (30)	CCS	China	80	63.3 ± 12.9	15/65	80	61.5 ± 10.3	19/61	miR-125b-5p	Blood	0.725	0.67602	0.64708	54	28	26	52
Wen 2022 (30)	CCS	China	80	63.3 ± 12.9	15/65	80	61.5 ± 10.3	19/61	miR-143-3p	Blood	0.751	0.6718	0.6914	54	25	26	55
Wen 2022 (30)	CCS	China	80	63.3 ± 12.9	15/65	80	61.5 ± 10.3	19/61	miR-182-5p	Blood	0.767	0.64311	0.73473	51	21	29	59
Wen 2022 (30)	CCS	China	80	63.3 ± 12.9	15/65	80	61.5 ± 10.3	19/61	miR-200c-3p	Blood	0.789	0.65694	0.73212	53	21	27	59
Miyake 2023 (31)	CCS	Japan	122	NA	19/103	10	NA	3/7	NMP-22	Urine	NA	0.52	1	63	0	59	10
Miyake 2023 (31)	CCS	Japan	122	NA	19/103	10	NA	3/7	VUC	Urine	NA	0.47	1	57	0	65	10
Guszcz 2023 (32)	CCS	Poland	92	70 (36–96)	26/67	26	67 (36–86)	13/13	Fibronectin	Plasma	0.98	0.97	0.84	89	4	3	18
Liu 2023 (33)	CCS	China	42	NA	9/33	42	NA	NA	Urinary cytology	Urine	0.597	0.214	0.976	9	1	33	41
Mamdouh 2023 (34)	CCS	Egypt	111	59.5 ± 7.6	12/99	25	55.4 ± 6.8	7/15	miR-200	Urine	0.854	0.622	1	69	0	42	25
Mamdouh 2023 (34)	CCS	Egypt	111	59.5 ± 7.6	12/99	25	55.4 ± 6.8	7/15	miR-145	Urine	0.886	0.784	0.917	87	2	24	23
Mamdouh 2023 (34)	CCS	Egypt	111	59.5 ± 7.6	12/99	25	55.4 ± 6.8	7/15	miR-21	Urine	0.89	0.838	1	93	0	18	25
Sequeira 2023 (35)	Cohort study	Portugal	73	70 (15)	12/61	74	46 (9)	27/47	hsa-miR-126-3p	Plasma	0.631	0.6301	0.6718	46	24	27	50
Sequeira 2023 (35)	Cohort study	Portugal	73	70 (15)	12/61	74	46 (9)	27/47	hsa-miR-182-5p	Plasma	0.67	0.6027	0.7333	44	20	29	54
Sequeira 2023 (35)	Cohort study	Portugal	73	70 (15)	12/61	74	46 (9)	27/47	hsa-miR-375-3p	Plasma	0.647	0.3973	0.8615	29	10	44	64
Yu 2023 (36)	CCS	China	112	61.8 ± 13.6	21/91	112	59.3 ± 13.1	27/85	miR-142-5p	Urine	0.614	0.8333	0.4762	93	59	19	53
Yu 2023 (36)	CCS	China	113	61.8 ± 13.7	21/92	113	59.3 ± 13.2	27/86	miR-223-3p	Urine	0.603	0.8095	0.3571	91	72	21	40
Yu 2023 (36)	CCS	China	114	61.8 ± 13.8	21/93	114	59.3 ± 13.3	27/87	miR-381-3p	Urine samples	0.723	0.7857	0.6071	88	44	24	68
Yu 2023 (36)	CCS	China	115	61.8 ± 13.9	21/94	115	59.3 ± 13.4	27/88	miR-451a	Urine	0.766	0.6786	0.75	76	28	36	84
Yu 2023 (36)	CCS	China	116	61.8 ± 13.10	21/95	116	59.3 ± 13.5	27/89	miR-27b-3p	Urine	0.664	0.369	0.9405	41	7	71	105
Yu 2023 (36)	CCS	China	117	61.8 ± 13.11	21/96	117	59.3 ± 13.6	27/90	miR-27b-3p/miR-381-3p/miR-451a	Urine	0.894	0.869	0.7738	97	25	15	87
Gayed 2024 (37)	CCS	Egypt	50	67.50 ± 99	12/38	50	68 ± 9.37	14/36	Micro RNA130a-3p	Blood	0.964	0.9	0.78	45	11	5	39
Gayed 2024 (37)	CCS	Egypt	51	67.50 ± 100	12/39	51	69 ± 9.37	14/37	Micro RNA301a-3p	Blood	0.973	0.92	0.96	46	2	4	48
Yang 2024 (38)	CCS	China	116	58 (27, 75)	47/69	116	57 (26, 78)	32/84	miR-146a-5p	Urine	0.6874	0.7242	0.5387	84	54	32	62
Yang 2024 (38)	CCS	China	117	59 (27, 75)	47/70	117	58 (26, 78)	32/85	miR-93-5p	Urine	0.63	0.58732	0.54642	68	53	48	63
Yang 2024 (38)	CCS	China	118	60 (27, 75)	47/71	118	59 (26, 78)	32/86	miR-663b	Urine	0.6086	0.54789	0.57113	64	50	52	66
Yang 2024 (38)	CCS	China	119	61 (27, 75)	47/72	119	60 (26, 78)	32/87	miR-21	Urine	0.8517	0.7901	0.7137	92	33	24	83
Yang 2024 (38)	CCS	China	120	62 (27, 75)	47/73	120	61 (26, 78)	32/88	miR-4454	Urine	0.7684	0.63986	0.78486	74	25	42	91
Yang 2024 (38)	CCS	China	121	63 (27, 75)	47/74	121	62 (26, 78)	32/89	Urine cytology	Urine	0.6422	0.45606	0.76696	53	27	63	89
Yang 2024 (38)	CCS	China	122	64 (27, 75)	47/75	122	63 (26, 78)	32/90	Emdp-miR	Urine	0.93	0.87986	0.85681	102	17	14	99

CCS, case–control study; ANG, angiogenin; F/M, female/male; AUC, area under the receiver operating characteristic curve; TP, true positive; FP, false positive; FN, false negative; TN, true negative; *TERT*, urine sediments; VUC, voided urine cytology; Emdp-miR panel, miR-146a-5p, miR-93-5p, miR-21, and miR-4454; NA, not available.

**Table 2:** Efficacy analysis of urine-derived biomarkers.

Biomarker	Sensitivity (95% CI)	Specificity (95% CI)	DOR (95% CI)	Superiority index (95% CI)	Relative sensitivity (95% CI)	Relative specificity (95% CI)	Rhat (Sensitivity)	Rhat (Specificity)
Angiogenin	0.80 (0.51, 0.95)	0.79 (0.32, 0.99)	91.32 (1.51, 601.08)	5.28 (0.14, 13.00)	1.87 (0.90, 3.86)	0.83 (0.34, 1.21)	1.00	1.00
MicroRNA	0.78 (0.69, 0.84)	0.79 (0.64, 0.89)	15.42 (5.73, 32.24)	3.26 (0.20, 9.00)	1.82 (0.99, 3.56)	0.83 (0.65, 1.16)	1.00	1.00
*cfNDA* (*TERT*)	0.48 (0.21, 0.76)	0.96 (0.71, 1.00)	317,102.49 (2.08, 98,894.84)	2.81 (0.14, 9.00)	1.00 (1.00, 1.00)	1.00 (1.00, 1.00)	1.00	1.00
Survivin	0.74 (0.46, 0.92)	0.66 (0.24, 0.96)	17.12 (0.75, 103.89)	2.04 (0.11, 9.00)	1.74 (0.81, 3.56)	0.70 (0.25, 1.09)	1.00	1.00
UCA1	0.70 (0.48, 0.86)	0.66 (0.31, 0.92)	7.78 (0.94, 32.42)	1.07 (0.11, 7.00)	1.64 (0.80, 3.36)	0.69 (0.33, 1.05)	1.00	1.00
Urine cytology	0.43 (0.35, 0.51)	0.93 (0.87, 0.96)	12.05 (4.66, 21.55)	0.76 (0.20, 3.00)	1.00 (0.54, 2.04)	0.98 (0.88, 1.32)	1.00	1.00
NMP22	0.55 (0.40, 0.68)	0.75 (0.51, 0.92)	4.99 (1.25, 14.35)	0.50 (0.09, 3.00)	1.30 (0.65, 2.66)	0.79 (0.53, 1.10)	1.00	1.00

**Table 3:** Urine microRNA biomarkers.

Biomarker	Sensitivity (95% CI)	Specificity (95% CI)	DOR (95% CI)	Superiority index (95% CI)	Relative sensitivity (95% CI)	Relative specificity (95% CI)	Rhat (sensitivity)	Rhat (specificity)
miR-125b	0.86 (0.55, 0.97)	0.63 (0.22, 0.90)	27.98 (1.06, 130.67)	10.97 (0.09, 31.00)	1.18 (0.71, 1.99)	0.95 (0.35, 1.91)	1.00	1.00
miR-200	0.56 (0.23, 0.85)	0.93 (0.54, 1.00)	937,296.14 (1.35, 162,947.10)	8.89 (0.05, 31.00)	0.77 (0.30, 1.53)	1.44 (0.72, 3.08)	1.00	1.01
miR-221	0.76 (0.42, 0.94)	0.71 (0.31, 0.93)	19.47 (0.92, 87.18)	7.86 (0.04, 31.00)	1.00 (1.00, 1.00)	1.00 (1.00, 1.00)	1.00	1.01
miR-183	0.78 (0.56, 0.91)	0.72 (0.41, 0.89)	14.94 (1.71, 53.68)	7.08 (0.09, 29.00)	1.07 (0.70, 1.82)	1.09 (0.58, 2.17)	1.00	1.00
miR-21	0.82 (0.67, 0.90)	0.69 (0.48, 0.83)	12.96 (3.04, 32.49)	6.59 (0.29, 25.00)	1.13 (0.82, 1.91)	1.06 (0.62, 2.19)	1.00	1.01
miR-516a-5p	0.67 (0.31, 0.91)	0.75 (0.33, 0.98)	23.14 (0.59, 145.65)	6.58 (0.03, 31.05)	0.92 (0.40, 1.75)	1.16 (0.43, 2.60)	1.00	1.00
miR-145	0.64 (0.41, 0.81)	0.82 (0.54, 0.96)	15.90 (1.46, 58.36)	5.00 (0.05, 23.00)	0.88 (0.52, 1.58)	1.26 (0.71, 2.62)	1.00	1.00
miR-96	0.56 (0.33, 0.76)	0.88 (0.61, 0.98)	20.09 (1.54, 75.38)	4.67 (0.06, 23.00)	0.77 (0.42, 1.33)	1.35 (0.85, 2.78)	1.00	1.00
miR-381-3p	0.74 (0.37, 0.94)	0.58 (0.18, 0.91)	12.11 (0.35, 79.28)	4.67 (0.03, 31.00)	1.02 (0.47, 1.86)	0.90 (0.24, 2.08)	1.00	1.00
miR-4454	0.64 (0.31, 0.88)	0.74 (0.34, 0.95)	13.08 (0.60, 67.06)	4.45 (0.04, 27.00)	0.88 (0.39, 1.64)	1.14 (0.46, 2.43)	1.00	1.00
miR-142-5p	0.78 (0.40, 0.95)	0.50 (0.11, 0.87)	10.34 (0.24, 55.83)	3.98 (0.03, 27.00)	1.07 (0.52, 1.90)	0.78 (0.15, 1.96)	1.00	1.00
miR-451a	0.64 (0.28, 0.90)	0.68 (0.26, 0.95)	11.91 (0.34, 69.97)	3.91 (0.03, 25.00)	0.89 (0.36, 1.65)	1.05 (0.35, 2.40)	1.00	1.00
miR-205	0.76 (0.44, 0.93)	0.56 (0.17, 0.87)	9.21 (0.39, 42.81)	3.56 (0.03, 25.00)	1.04 (0.57, 1.81)	0.85 (0.26, 1.81)	1.00	1.00
miR-126	0.86 (0.58, 0.97)	0.41 (0.08, 0.77)	9.84 (0.31, 48.44)	3.52 (0.04, 21.00)	1.18 (0.75, 1.95)	0.61 (0.11, 1.47)	1.00	1.00
miR-146a-5p	0.71 (0.34, 0.91)	0.58 (0.20, 0.88)	7.33 (0.30, 35.08)	2.80 (0.03, 23.00)	0.97 (0.44, 1.74)	0.89 (0.28, 2.05)	1.00	1.00
miR-223-3p	0.76 (0.41, 0.94)	0.43 (0.09, 0.84)	6.74 (0.17, 39.70)	2.41 (0.03, 21.00)	1.05 (0.52, 1.88)	0.67 (0.12, 1.78)	1.00	1.00
miR-27b-3p	0.41 (0.12, 0.75)	0.85 (0.43, 0.99)	18.10 (0.25, 116.21)	2.18 (0.03, 17.05)	0.56 (0.16, 1.25)	1.31 (0.57, 2.77)	1.00	1.00
miR-210	0.67 (0.32, 0.90)	0.60 (0.19, 0.89)	6.59 (0.30, 30.60)	2.06 (0.03, 19.00)	0.92 (0.43, 1.61)	0.90 (0.28, 1.95)	1.00	1.00
miR-93-5p	0.64 (0.41, 0.82)	0.69 (0.40, 0.89)	6.05 (0.84, 21.74)	1.82 (0.03, 15.00)	0.89 (0.51, 1.62)	1.06 (0.51, 2.32)	1.00	1.00
miR-663b	0.58 (0.26, 0.85)	0.60 (0.20, 0.90)	4.30 (0.19, 21.39)	1.19 (0.03, 13.05)	0.79 (0.33, 1.52)	0.92 (0.27, 2.13)	1.00	1.00

**Table 4:** Urine-based combined diagnosis.

Biomarker	Sensitivity (95% CI)	Specificity (95% CI)	DOR (95% CI)	Superiority index (95% CI)	Relative sensitivity (95% CI)	Relative specificity (95% CI)	Rhat (sensitivity)	Rhat (specificity)
*TERT*/*FGFR3*/*TP53*/*PIK3CA*/*KRAS* (urine supernatant)	0.78 (0.32, 0.98)	0.85 (0.41, 0.99)	217.51 (1.24, 1,582.28)	8.54 (0.05, 27.00)	1.07 (0.56, 2.04)	1.14 (0.63, 2.11)	1.01	1.01
4 miRs-125b/145/183/221 + VUC	0.73 (0.33, 0.96)	0.85 (0.41, 0.99)	154.57 (1.12, 1,110.35)	7.24 (0.06, 27.00)	1.05 (0.41, 2.47)	1.17 (0.51, 2.56)	1.00	1.00
miR-96/miR-183	0.79 (0.33, 0.99)	0.77 (0.32, 0.97)	130.44 (0.78, 1,277.54)	5.69 (0.05, 23.00)	1.13 (0.43, 2.77)	1.06 (0.40, 2.33)	1.01	1.00
Emdp-miR	0.79 (0.34, 0.97)	0.77 (0.32, 0.97)	68.46 (0.88, 457.49)	5.18 (0.04, 27.00)	1.13 (0.42, 2.76)	1.06 (0.39, 2.26)	1.00	1.02
6 miRs-96/125b/126/145/183/221 + VUC	0.71 (0.25, 0.96)	0.83 (0.37, 0.99)	83.35 (0.72, 742.50)	4.61 (0.04, 25.00)	1.02 (0.28, 2.41)	1.14 (0.48, 2.45)	1.00	1.01
*TERT*/*FGFR3*/*TP53*/*HRAS*/*PIK3CA*/*KRAS*/*ERBB*2 (urine sediments)	0.77 (0.30, 0.97)	0.79 (0.34, 0.98)	75.46 (0.72, 481.11)	4.52 (0.04, 25.00)	1.00 (1.00, 1.00)	1.00 (1.00, 1.00)	1.00	1.01
miR-96/cytology	0.72 (0.29, 0.96)	0.81 (0.36, 0.98)	65.28 (0.77, 436.91)	4.39 (0.04, 25.00)	1.04 (0.37, 2.63)	1.12 (0.47, 2.41)	1.00	1.01
4 miRs-125b/145/183/221	0.65 (0.26, 0.92)	0.86 (0.44, 0.99)	88.03 (0.88, 591.38)	4.04 (0.05, 21.00)	0.93 (0.34, 2.33)	1.18 (0.53, 2.52)	1.00	1.01
miR-183/cytology	0.69 (0.27, 0.95)	0.81 (0.33, 0.98)	54.62 (0.65, 360.32)	3.85 (0.04, 23.00)	1.00 (0.34, 2.54)	1.11 (0.43, 2.36)	1.00	1.01
miR-93-5p/miR-516a-5p	0.77 (0.28, 0.97)	0.74 (0.30, 0.96)	51.04 (0.43, 266.49)	3.74 (0.04, 23.00)	1.11 (0.35, 2.69)	1.02 (0.37, 2.26)	1.01	1.00
miR-27b-3p/miR-381-3p/miR-451a	0.77 (0.27, 0.97)	0.70 (0.28, 0.96)	38.21 (0.43, 236.36)	3.39 (0.04, 23.00)	1.11 (0.35, 2.61)	0.97 (0.34, 2.21)	1.00	1.00
6 miRs-96/125b/126/145/183/221	0.66 (0.23, 0.93)	0.82 (0.37, 0.99)	46.39 (0.57, 289.68)	2.79 (0.04, 19.00)	0.95 (0.30, 2.33)	1.13 (0.47, 2.43)	1.00	1.01
Survivin/NMP22/cytology	0.70 (0.29, 0.95)	0.75 (0.30, 0.96)	30.27 (0.51, 210.40)	2.52 (0.04, 19.00)	1.01 (0.37, 2.49)	1.03 (0.38, 2.22)	1.00	1.02
Survivin/cytology	0.70 (0.28, 0.96)	0.75 (0.30, 0.97)	27.54 (0.55, 152.31)	2.46 (0.04, 21.00)	1.01 (0.36, 2.51)	1.03 (0.37, 2.24)	1.00	1.01
NMP22/cytology	0.57 (0.18, 0.90)	0.70 (0.28, 0.95)	10.32 (0.26, 59.47)	0.78 (0.03, 7.00)	0.82 (0.24, 2.17)	0.97 (0.34, 2.17)	1.00	1.01

**Table 5:** Efficacy analysis of blood-derived biomarkers.

Biomarker	Sensitivity (95% CI)	Specificity (95% CI)	DOR (95% CI)	Superiority index (95% CI)	Relative sensitivity (95% CI)	Relative specificity (95% CI)	Rhat (sensitivity)	Rhat (specificity)
miR-181b-5p/miR-183-5p/miR-199-5p/miR-211-3p	0.80 (0.43, 0.96)	0.91 (0.63, 0.99)	190.07 (4.16, 960.66)	3.02 (0.20, 7.00)	1.77 (0.78, 4.79)	0.93 (0.66, 1.09)	1.00	1.00
Fibronectin	0.93 (0.65, 0.99)	0.76 (0.34, 0.96)	283.17 (2.54, 1,671.68)	3.02 (0.14, 7.00)	2.06 (0.99, 5.84)	0.78 (0.36, 1.01)	1.02	1.00
miR-132-3p/miR-7-5p/miR-148b-3p	0.85 (0.57, 0.97)	0.79 (0.52, 0.94)	55.49 (3.26, 280.71)	1.82 (0.14, 7.00)	1.90 (0.91, 5.76)	0.81 (0.53, 1.00)	1.00	1.01
Angiogenin	0.54 (0.16, 0.89)	0.98 (0.86, 1.00)	113,658.88 (3.45, 272,255.94)	1.63 (0.20, 7.00)	1.00 (1.00, 1.00)	1.00 (1.00, 1.00)	1.02	1.00
MicroRNA	0.74 (0.66, 0.80)	0.76 (0.69, 0.82)	9.65 (5.21, 15.82)	0.34 (0.11, 1.00)	1.65 (0.77, 4.86)	0.78 (0.70, 0.92)	1.01	1.00

### 3.2 Quality assessments

**Figure 2:**
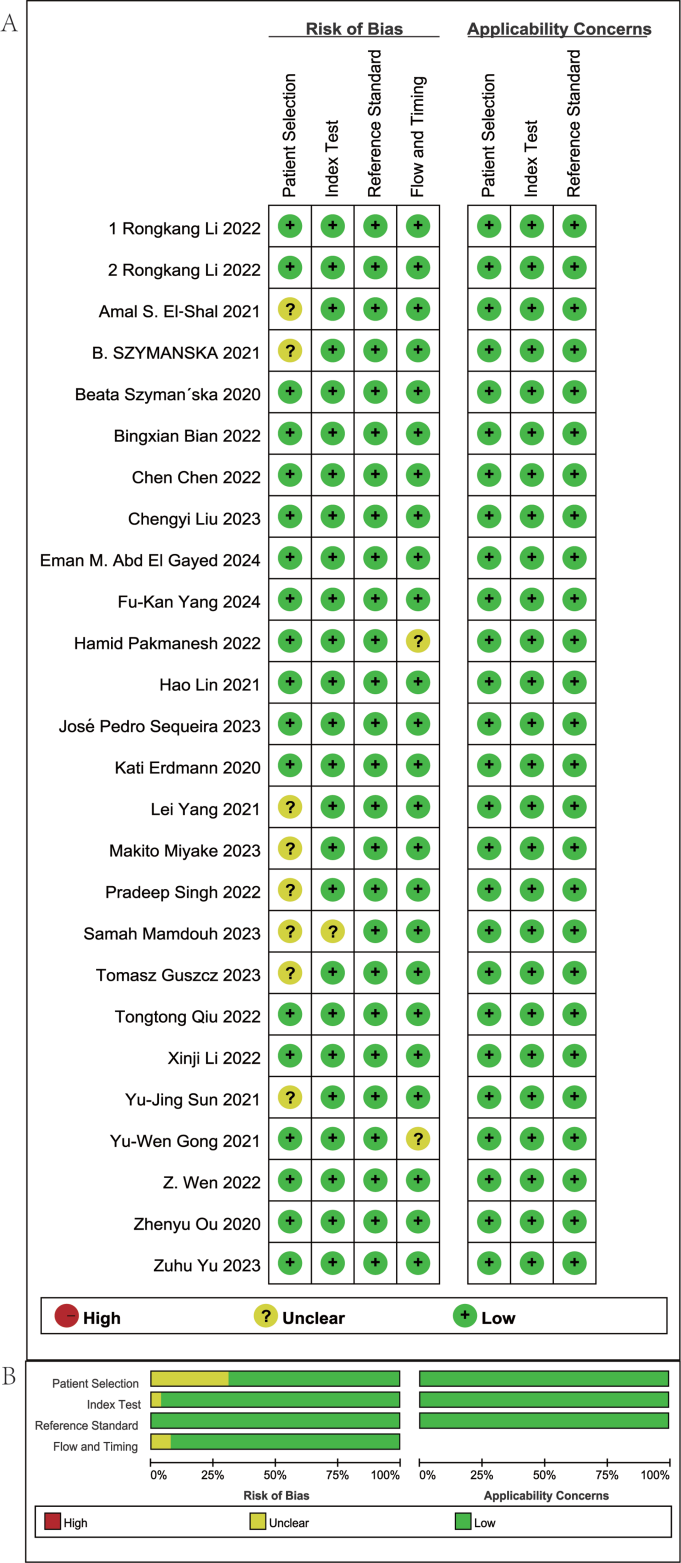
Summary of risk of bias in the studies included. **(A)** Risk of bias in the studies included. **(B)** Summary of risk of bias in the studies included.

The methodological rigor of the 26 eligible studies was evaluated with the QUADAS-2 instrument, encompassing four aspects: patient selection, index evaluation, reference criterion, and flow/timing. The results of the quality assessment are shown in **Figure 2**. Within the Patient Selection domain, 10 studies were judged to carry an uncertain risk of bias, while the others were deemed low risk. This suggests that some studies did not clearly describe their selection methods or may have involved selective enrollment. In the Index Test and Reference Standard domains, all studies were judged to have a low risk of bias, indicating a consistent application of diagnostic tests and reference standards. In the Flow and Timing domain, 25 studies were assessed as having minimal bias, while three were rated as unclear, primarily due to insufficient reporting on the interval between the index test and the reference method or incomplete analytical workflows. Overall, the included studies demonstrated high methodological quality with respect to index tests, reference standards, and process control, although some information gaps concerning participant enrollment remained.

### 3.3 Efficacy analysis of urine-derived biomarkers

**Figure 3:**
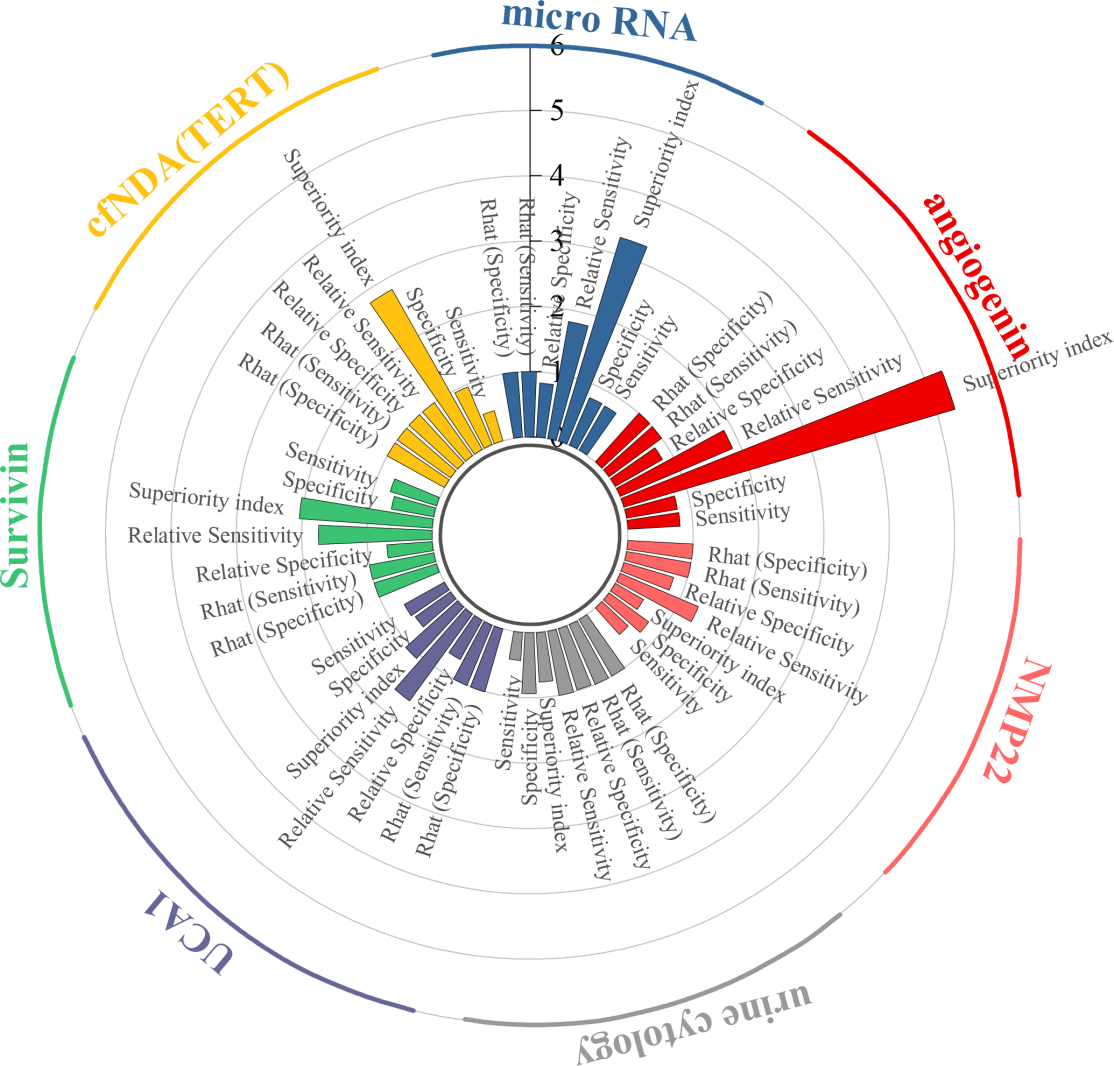
Efficacy analysis of urine-derived biomarkers.

This study evaluated the diagnostic efficacy of seven urine-derived biomarkers for bladder cancer. For angiogenin, the pooled sensitivity was 0.80 (95% CI: 0.51–0.95), specificity was 0.79 (95% CI: 0.32–0.99), and the superiority index was 5.28 (95% CI: 0.14–13.00). For microRNAs, the pooled sensitivity and specificity were 0.78 (95% CI: 0.69–0.84) and 0.79 (95% CI: 0.64–0.89), with a superiority index of 3.26 (95% CI: 0.20–9.00). For *cfDNA* (*TERT*), sensitivity was 0.48 (95% CI: 0.21–0.76) and specificity was 0.96 (95% CI: 0.71–1.00), with a superiority index of 2.81 (95% CI: 0.14–9.00). For survivin, the pooled sensitivity and specificity were 0.74 (95% CI: 0.46–0.92) and 0.66 (95% CI: 0.24–0.96), with a superiority index of 2.04 (95% CI: 0.11–9.00). For UCA1, sensitivity and specificity were 0.70 (95% CI: 0.48–0.86) and 0.66 (95% CI: 0.31–0.92), with a superiority index of 1.07 (95% CI: 0.11–7.00). Urine cytology demonstrated lower sensitivity at 0.43 (95% CI: 0.35–0.51) but higher specificity at 0.93 (95% CI: 0.80–0.96), with a superiority index of 0.76 (95% CI: 0.20–3.00). Finally, NMP22 showed moderate diagnostic performance, with sensitivity of 0.55 (95% CI: 0.40–0.68), specificity of 0.75 (95% CI: 0.51–0.92), and a superiority index of 0.50 (95% CI: 0.09–3.00). Detailed results are presented in **Table 2** and **Figure 3**.

### 3.4 Urine micro-RNA biomarker

This study evaluated the diagnostic performance of 20 different urinary microRNAs in bladder cancer. For miR-125b, the pooled sensitivity was 0.86 (95% CI: 0.55–0.97), the specificity was 0.63 (95% CI: 0.22–0.90), and the superiority index was 10.97 (95% CI: 0.09–31.00). For miR-200, sensitivity and specificity were 0.56 (95% CI: 0.23–0.85) and 0.93 (95% CI: 0.54–1.00), respectively, with a superiority index of 8.89 (95% CI: 0.05–31.00). For miR-221, sensitivity was 0.76 (95% CI: 0.42–0.94) and specificity was 0.71 (95% CI: 0.31–0.93), with a superiority index of 7.86 (95% CI: 0.04–31.00). For miR-183, sensitivity was 0.78 (95% CI: 0.56–0.91) and specificity was 0.72 (95% CI: 0.41–0.89), with a superiority index of 7.08 (95% CI: 0.09–29.00). Finally, for miR-21, the pooled sensitivity was 0.82 (95% CI: 0.67–0.90), the specificity was 0.69 (95% CI: 0.48–0.83), and the superiority index was 6.59 (95% CI: 0.29–25.00). Detailed results for all microRNAs are presented in **Table 3**.

### 3.5 Urine-based combined diagnosis

This study evaluated the diagnostic performance of several combined urine-based biomarkers for the non-invasive detection of bladder cancer. For the biomarker panel *TERT/FGFR3/TP53/PIK3CA/KRAS*, the pooled sensitivity was 0.78 (95% CI: 0.32–0.98), the specificity was 0.85 (95% CI: 0.41–0.99), and the superiority index was 8.54 (95% CI: 0.05–27.00). For the combination of four microRNAs (miR-125b/145/183/221) with VUC, sensitivity was 0.73 (95% CI: 0.33–0.96), specificity was 0.85 (95% CI: 0.41–0.99), and superiority index was 7.24 (95% CI: 0.06–27.00). The combination of miR-96 and miR-183 achieved sensitivity of 0.79 (95% CI: 0.33–0.99), specificity of 0.77 (95% CI: 0.32–0.97), and a superiority index of 5.69 (95% CI: 0.05–23.00). The Emdp-miR panel (miR-146a-5p, miR-93-5p, miR-21, and miR-4454) showed sensitivity of 0.79 (95% CI: 0.34–0.97), specificity of 0.77 (95% CI: 0.32–0.97), and a superiority index of 5.18 (95% CI: 0.04–27.00). Lastly, the six-miRNA panel (miR-96/125b/126/145/183/221) combined with VUC demonstrated sensitivity of 0.71 (95% CI: 0.25–0.96), specificity of 0.83 (95% CI: 0.37–0.99), and a superiority index of 4.61 (95% CI: 0.04–25.00). Detailed results are presented in **Table 4**.

### 3.6 Efficacy analysis of blood-derived biomarkers

**Figure 4:**
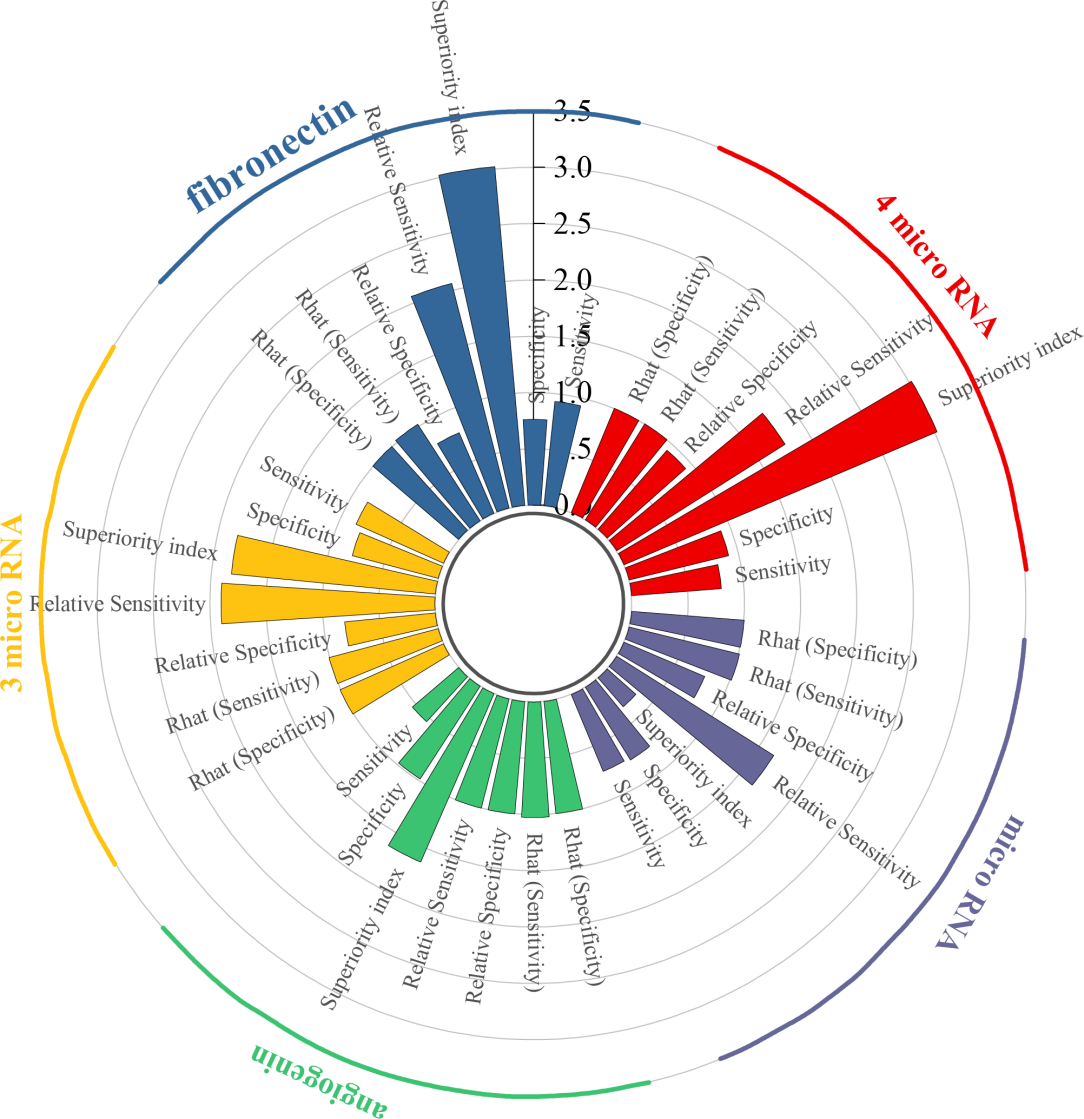
Efficacy analysis of blood-derived biomarkers.

To begin with, the biomarker panel composed of miR-181b-5p, miR-183-5p, miR-199-5p, and miR-211-3p demonstrated a pooled sensitivity of 0.80 (95% CI: 0.43–0.96), specificity of 0.91 (95% CI: 0.63–0.99), and a superiority index of 3.02 (95% CI: 0.20–7.00). Similarly, fibronectin exhibited high sensitivity at 0.93 (95% CI: 0.65–0.99) and specificity of 0.76 (95% CI: 0.34–0.96), with a superiority index of 3.02 (95% CI: 0.14–7.00). In addition, the combined panel of miR-132-3p, miR-7-5p, and miR-148b-3p showed a sensitivity of 0.85 (95% CI: 0.57–0.97), specificity of 0.79 (95% CI: 0.52–0.94), and a superiority index of 1.82 (95% CI: 0.14–7.00). For angiogenin, sensitivity was comparatively low at 0.54 (95% CI: 0.16–0.89), whereas specificity was exceptionally high at 0.98 (95% CI: 0.86–1.00), with a superiority index of 1.63 (95% CI: 0.20–7.00). When microRNAs were considered as a broader diagnostic category, pooled sensitivity was 0.74 (95% CI: 0.66–0.80), specificity was 0.76 (95% CI: 0.69–0.82), and the superiority index was 0.34 (95% CI: 0.11–1.00). Detailed results are presented in **Table 5** and **Figure 4**.

### 3.7 Blood-based microRNA biomarker

The biomarker microRNA-301a-3p exhibited strong diagnostic potential, with a sensitivity of 0.87 (95% CI: 0.53–0.98), specificity of 0.92 (95% CI: 0.64–1.00), and a superiority index of 50.71 (95% CI: 2.33–63.00). MicroRNA-130a-3p also demonstrated favorable accuracy, with sensitivity of 0.84 (95% CI: 0.50–0.98), specificity of 0.73 (95% CI: 0.33–0.94), and a superiority index of 18.31 (95% CI: 0.10–59.00). For miR-148b-3p, sensitivity was 0.76 (95% CI: 0.41–0.95), specificity was 0.67 (95% CI: 0.33–0.91), and the superiority index was 8.89 (95% CI: 0.06–53.00). The performance of miR-10a-5p was also noteworthy, with sensitivity of 0.75 (95% CI: 0.37–0.94), specificity of 0.65 (95% CI: 0.29–0.91), and a superiority index of 8.22 (95% CI: 0.02–55.00). Finally, miR-203 showed sensitivity of 0.73 (95% CI: 0.37–0.94), specificity of 0.69 (95% CI: 0.30–0.92), and a superiority index of 7.13 (95% CI: 0.03–53.00). Detailed results are presented in **Table 6**.

**Table 6:** Blood microRNA biomarkers.

Biomarker	Sensitivity (95% CI)	Specificity (95% CI)	DOR (95% CI)	Superiority index (95% CI)	Relative sensitivity (95% CI)	Relative specificity (95% CI)	Rhat (sensitivity)	Rhat (specificity)
Micro RNA301a-3p	0.87 (0.53, 0.98)	0.92 (0.64, 1.00)	621.45 (7.33, 3,822.16)	50.71 (2.33, 63.00)	1.61 (0.79, 3.58)	1.61 (0.92, 3.20)	1.02	1.01
Micro RNA130a-3p	0.84 (0.50, 0.98)	0.73 (0.33, 0.94)	46.98 (1.39, 258.76)	18.31 (0.10, 59.00)	1.56 (0.68, 3.49)	1.27 (0.50, 2.60)	1.02	1.01
miR-148b-3p	0.76 (0.41, 0.95)	0.67 (0.33, 0.91)	16.38 (0.71, 81.10)	8.89 (0.06, 53.00)	1.39 (0.61, 2.97)	1.18 (0.50, 2.48)	1.00	1.01
miR-10a-5p	0.75 (0.37, 0.94)	0.65 (0.29, 0.91)	14.96 (0.56, 74.72)	8.22 (0.02, 55.00)	1.40 (0.51, 3.03)	1.15 (0.41, 2.51)	1.01	1.00
miR-203	0.73 (0.37, 0.94)	0.69 (0.30, 0.92)	15.81 (0.70, 76.65)	7.13 (0.03, 53.00)	1.35 (0.57, 3.11)	1.21 (0.47, 2.50)	1.00	1.00
miR-34a	0.71 (0.35, 0.93)	0.69 (0.29, 0.92)	14.49 (0.61, 75.08)	6.39 (0.02, 51.00)	1.32 (0.52, 2.97)	1.20 (0.44, 2.65)	1.00	1.01
miR-132-3p	0.63 (0.27, 0.88)	0.76 (0.44, 0.95)	12.77 (0.65, 58.96)	6.30 (0.03, 43.00)	1.17 (0.44, 2.67)	1.33 (0.67, 2.78)	1.01	1.01
miR-9	0.71 (0.37, 0.94)	0.67 (0.29, 0.92)	14.09 (0.55, 74.04)	6.13 (0.02, 51.00)	1.33 (0.55, 3.15)	1.17 (0.41, 2.49)	1.01	1.00
miR-7-5p	0.57 (0.25, 0.85)	0.78 (0.45, 0.95)	11.46 (0.53, 59.46)	5.19 (0.02, 45.00)	1.05 (0.39, 2.49)	1.37 (0.65, 2.82)	1.01	1.01
hsa-miR-182-5p	0.64 (0.37, 0.84)	0.75 (0.50, 0.90)	8.29 (1.00, 26.11)	4.56 (0.10, 35.00)	1.14 (0.70, 2.04)	1.29 (0.81, 2.32)	1.00	1.02
miR-181b-5p	0.62 (0.28, 0.87)	0.73 (0.40, 0.93)	9.07 (0.55, 43.81)	4.55 (0.03, 37.00)	1.14 (0.45, 2.66)	1.28 (0.60, 2.61)	1.01	1.00
miR-100-5p	0.68 (0.33, 0.90)	0.66 (0.32, 0.90)	8.45 (0.51, 35.37)	4.19 (0.02, 31.05)	1.26 (0.48, 2.76)	1.15 (0.46, 2.43)	1.02	1.00
miR-183-5p	0.65 (0.32, 0.89)	0.69 (0.36, 0.91)	7.99 (0.56, 36.43)	3.84 (0.02, 37.00)	1.20 (0.50, 2.70)	1.21 (0.52, 2.63)	1.00	1.00
miR-9-5p	0.65 (0.32, 0.90)	0.68 (0.35, 0.90)	7.95 (0.59, 36.75)	3.72 (0.03, 29.00)	1.21 (0.50, 2.75)	1.20 (0.51, 2.54)	1.00	1.01
miR-200c-3p	0.63 (0.29, 0.87)	0.70 (0.33, 0.91)	7.78 (0.47, 35.46)	3.72 (0.02, 31.00)	1.17 (0.46, 2.68)	1.22 (0.50, 2.53)	1.01	1.01
hsa-miR-375-3p	0.45 (0.17, 0.78)	0.83 (0.55, 0.96)	8.80 (0.62, 38.48)	3.51 (0.03, 35.00)	0.81 (0.29, 1.74)	1.44 (0.83, 2.67)	1.00	1.02
hsa-miR-126-3p	0.63 (0.31, 0.88)	0.67 (0.35, 0.89)	6.66 (0.55, 29.98)	3.16 (0.02, 25.15)	1.14 (0.53, 2.30)	1.16 (0.56, 2.23)	1.00	1.02
miR-182-5p	0.59 (0.27, 0.87)	0.69 (0.35, 0.91)	6.48 (0.45, 30.17)	2.72 (0.02, 17.00)	1.10 (0.40, 2.50)	1.21 (0.50, 2.50)	1.00	1.00
miR-199a-5p	0.71 (0.39, 0.92)	0.57 (0.24, 0.84)	6.48 (0.47, 30.02)	2.64 (0.02, 15.68)	1.32 (0.62, 2.89)	1.00 (0.37, 2.29)	1.01	1.00
miR-143-3p	0.64 (0.29, 0.88)	0.65 (0.31, 0.89)	6.21 (0.40, 26.39)	2.51 (0.02, 16.33)	1.18 (0.43, 2.64)	1.14 (0.46, 2.45)	1.00	1.00
miR-125b-5p	0.65 (0.30, 0.91)	0.62 (0.28, 0.88)	6.01 (0.37, 25.28)	2.25 (0.02, 13.68)	1.20 (0.44, 2.65)	1.08 (0.41, 2.30)	1.02	1.00
hsa-miR-34a-5p	0.65 (0.31, 0.89)	0.61 (0.28, 0.86)	5.32 (0.41, 22.14)	2.22 (0.02, 15.00)	1.15 (0.64, 2.05)	1.03 (0.53, 1.83)	1.00	1.00
hsa-miR-1-3p	0.73 (0.38, 0.92)	0.50 (0.20, 0.80)	4.99 (0.29, 22.01)	2.00 (0.02, 13.67)	1.28 (0.77, 2.19)	0.85 (0.41, 1.61)	1.00	1.00
miR-145-5p	0.70 (0.35, 0.92)	0.54 (0.23, 0.84)	5.74 (0.34, 27.07)	1.99 (0.02, 13.67)	1.29 (0.57, 2.85)	0.95 (0.35, 2.18)	1.01	1.00
miR-30a-5p	0.68 (0.35, 0.90)	0.56 (0.24, 0.84)	4.98 (0.39, 24.18)	1.78 (0.02, 13.00)	1.26 (0.53, 2.83)	0.98 (0.37, 2.15)	1.00	1.00
hsa-miR-23b-3p	0.68 (0.34, 0.89)	0.54 (0.25, 0.81)	4.53 (0.34, 19.81)	1.72 (0.02, 11.67)	1.21 (0.68, 2.04)	0.92 (0.48, 1.77)	1.00	1.00
miR-1-3p	0.66 (0.31, 0.88)	0.57 (0.24, 0.84)	4.72 (0.30, 19.13)	1.59 (0.02, 10.33)	1.22 (0.49, 2.78)	1.00 (0.36, 2.23)	1.01	1.00
hsa-miR-124-3p	0.55 (0.24,0.83)	0.67 (0.35, 0.90)	4.40 (0.32, 18.31)	1.58 (0.02, 11.00)	0.96 (0.50,1.76)	1.14 (0.63,2.10)	1.01	1.00
hsa-miR-196a-5p	0.60 (0.26,0.85)	0.62 (0.28, 0.86)	4.21 (0.30, 18.67)	1.50 (0.02, 10.33)	1.00 (1.00,1.00)	1.00 (1.00,1.00)	1.00	1.01
miR-106a-5p	0.67 (0.32,0.91)	0.51 (0.22, 0.84)	4.62 (0.30, 24.09)	1.40 (0.02, 10.60)	1.24 (0.51,2.76)	0.90 (0.32,2.05)	1.01	1.01
let-7c-5p	0.56 (0.24,0.84)	0.65 (0.29, 0.89)	4.39 (0.32, 20.46)	1.29 (0.02, 8.61)	1.05 (0.37,2.59)	1.14 (0.44,2.44)	1.00	1.01
miR-221-3p	0.54 (0.22, 0.83)	0.65 (0.29, 0.89)	4.01 (0.28, 16.99)	1.06 (0.02, 6.43)	1.02 (0.36, 2.56)	1.13 (0.43, 2.44)	1.00	1.01

### 3.8 ANOVA model for NMA

Results from the ANOVA-based network meta-analysis indicated that among urine-derived biomarkers, angiogenin exhibited the highest superiority index at 5.28 (95% CI: 0.14–13.00). Within the urinary microRNA subgroup, miR-125b demonstrated the greatest diagnostic advantage, with a superiority index of 10.97 (95% CI: 0.09–31.00). For combined urinary biomarkers, the *TERT*/*FGFR3*/*TP53*/*PIK3CA*/*KRAS* panel (urine supernatant-based) ranked highest, with a superiority index of 8.54 (95% CI: 0.05–27.00). In the category of blood-derived biomarkers, both the combined panel of miR-181b-5p/miR-183-5p/miR-199-5p/miR-211-3p and fibronectin exhibited the highest superiority indices, with values of 3.02 (95% CI: 0.20–7.00) and 3.02 (95% CI: 0.14–7.00), respectively. Finally, within the blood microRNA subgroup, microRNA-301a-3p showed the strongest diagnostic performance overall, with a superiority index of 50.71 (95% CI: 2.33–63.00).

### 3.9 Model fit quality and Rhat assessment in Bayesian analysis

In the Bayesian network meta-analysis conducted in this study, the Rhat values for both sensitivity and specificity across all models were close to 1, indicating satisfactory convergence of the MCMC simulations and stability of the posterior distributions. These findings suggest that the constructed Bayesian models achieved adequate fit and statistical stability, thereby ensuring the reliability of the estimated diagnostic performance parameters and the robustness of subsequent analytical outcomes.

## 4 Discussion

### 4.1 Key findings and potential reasons

In this study, significant differences were observed in the diagnostic performance of various types of non-invasive biomarkers for bladder cancer, including those derived from both urine and blood. These differences were evident across multiple comparative dimensions, such as single versus combined biomarkers, nucleic acid-based versus protein-based markers, and liquid biopsy samples. A total of 26 primary studies were included to establish the evidence base for this Bayesian network meta-analysis. Specifically, seven categories of urine-based biomarkers and five categories of blood-based biomarkers were evaluated. In addition, subgroup analyses were conducted for microRNAs from both urine and blood, comprising 20 and 32 individual microRNAs, respectively, and 15 combined diagnostic strategies were assessed. These findings highlight the heterogeneity in diagnostic performance across different biomarker sources and types. Notable differences in diagnostic efficacy were revealed between biomarker origins. Among urine-derived biomarkers, angiogenin demonstrated the highest superiority index. Within the urinary microRNA subgroup, miR-125b exhibited the strongest diagnostic potential. For combined diagnostic panels, the *TERT*/*FGFR3*/*TP53*/*PIK3CA*/*KRAS* panel (urine supernatant-based) showed the best performance, suggesting that multi-marker combinations may further enhance diagnostic accuracy. Among blood-derived biomarkers, the panel comprising miR-181b-5p, miR-183-5p, miR-199-5p, and miR-211-3p together with fibronectin showed the highest diagnostic efficacy. Notably, within the blood microRNA subgroup, microRNA-301a-3p achieved the highest superiority index, representing the most promising biomarker among all candidates included in this study.

### 4.2 Angiogenin

Angiogenin, belonging to the ribonuclease A family, has been reported to enhance angiogenesis and metastatic progression in bladder cancer through the activation of critical downstream elements within the PI3K–AKT–mTOR pathway (40). Angiogenesis supports the growth and survival of bladder tumors by supplying essential nutrients and oxygen while simultaneously altering the tumor microenvironment to facilitate cancer cell invasion and metastasis, thereby driving tumor initiation and progression (41). In addition, angiogenin suppresses the expression of DNA methyltransferase 3b (DNMT3b), resulting in hypomethylation of the matrix metalloproteinase-2 (MMP2) gene promoter and subsequent activation of MMP2 expression. The MMP2 protein promotes extracellular matrix degradation, thereby enhancing bladder cancer cell invasion and metastasis and contributing to disease progression (42). Aalami and colleagues conducted a comprehensive systematic review and meta-analysis including 1,051 subjects, which demonstrated a pooled sensitivity of 70.1% and a specificity of 78.7% for urinary angiogenin, underscoring its diagnostic reliability and clinical applicability in bladder cancer detection through non-invasive methods. Taken together, these findings suggest that angiogenin functions not only as a key molecular regulator in bladder tumorigenesis and progression but also as a promising biomarker for early screening or as an adjunct tool to improve non-invasive urological diagnostic strategies (43).

### 4.3 miR-125b

In bladder cancer, miR-125b functions as a tumor suppressor by post-transcriptionally regulating IL-6R and STAT3 through direct interaction with their 3′ untranslated regions (3′-UTRs). This regulation suppresses IL-6/IL-6R/STAT3 signaling activity, leading to reduced proliferation, migration, and invasiveness of cancer cells. At the same time, miR-125b increases the levels of apoptotic effectors such as Bax and cleaved caspase-3 while decreasing the expression of anti-apoptotic molecules such as Bcl-2, thereby promoting programmed cell death (44). Furthermore, Amuran and colleagues conducted urinary miRNA profiling to compare bladder cancer patients with healthy individuals, revealing that miR-125b expression was markedly reduced in the cancer group. Among the evaluated candidates, miR-125b demonstrated the highest diagnostic accuracy, achieving an AUC value of 0.801 and a specificity of 95.65%, underscoring its strong potential as a non-invasive biomarker in clinical practice (45).

### 4.4 Fibronectin

Fibronectin exhibits context-dependent behavior in oncogenesis. During the initial phases of tumorigenesis, its expression is often downregulated, thereby facilitating the bypass of cellular senescence and impairing early tumor-suppressive mechanisms. In contrast, at later stages, fibronectin is re-expressed at high levels, promoting tumor cell invasion, metastasis, and immune evasion. Moreover, fibronectin deposition within the tumor microenvironment supports tumor growth and angiogenesis, while hypoxia-induced re-expression further enhances the metastatic potential and drug resistance of tumor cells (46). Fibronectin also promotes cancer cell survival by activating the FAK/Src signaling cascade, thereby stimulating proliferation and reducing apoptosis through increased NF-κB activity and suppression of p21 expression (47). In a meta-analysis conducted by Dong et al. in 2018, it was reported that urinary fibronectin detection exhibited sensitivity of 80%, specificity of 79%, and an AUC value of 0.83, suggesting its potential as a promising non-invasive diagnostic biomarker for bladder cancer (48).

### 4.5 microRNA-301a-3p

MicroRNA-301a-3p, a member of the miRNA-130 family, has been shown to promote the proliferation of various cancer cells by targeting *PTEN* and activating oncogenic signaling pathways (49). Previous studies have demonstrated that microRNA-301a-3p facilitates tumor progression by suppressing *NKRF*, thereby activating the NF-κB signaling pathway and upregulating downstream effectors such as *MMP-2*, *MMP-9*, and *VEGF*. This activation enhances tumor cell proliferation, invasion, and migration (50). In a study conducted by Wang et al. in 2020, microRNA-301a-3p was found to be significantly upregulated in the serum of bladder cancer patients, achieving an AUC values of 0.892, which highlights its strong diagnostic accuracy and potential as a clinically relevant biomarker (49).

### 4.6 miR-200

The miR-200 family exerts diverse and context-dependent functions in cancer. These microRNAs primarily regulate cellular growth, motility, tissue infiltration, and apoptosis by targeting key genes such as *CDK6*, *AKT2*, *PTEN*, and *ZEB1/2*—for example, the miR-200 family suppresses epithelial–mesenchymal transition (EMT) by targeting ZEB1 and ZEB2, thereby reducing the migratory and invasive capacities of tumor cells. In addition, they influence apoptosis and immune responses by suppressing anti-apoptotic genes such as *XIAP* and immune checkpoint molecules such as PD-L1 (51). Elevated levels of miR-200a were found to downregulate Dicer, consequently impairing the maturation of miR-16. As a result, the reduced levels of mature miR-16 diminish its ability to inhibit *JNK2* translation, leading to elevated *JNK2* protein expression. The upregulation of *JNK2* subsequently promotes the phosphorylation of c-Jun, which, in turn, enhances the transcription of the *MMP-2* gene. As a matrix metalloproteinase, *MMP-2* degrades the extracellular matrix, thereby increasing the invasive capacity of bladder cancer cells (52).

### 4.7 Survivin

Survivin, a potent inhibitor of apoptosis belonging to the inhibitor of apoptosis protein (IAP) family, typically does not appear in normal tissues but is highly expressed in most malignant tumors. This expression profile suggests that survivin could act as a biomarker to aid in the early detection of bladder cancer (53). Survivin contributes to bladder cancer progression through multiple mechanisms. It inhibits apoptosis, enabling cancer cells to evade intrinsic death signaling; regulates cell cycle progression, especially at the G2/M transition point, thereby promoting uncontrolled proliferation; and is associated with chemoresistance by suppressing chemotherapy-induced apoptosis, ultimately reducing the efficacy of anticancer therapies (54). Collectively, these mechanisms drive tumor initiation, progression, and the development of treatment resistance in bladder cancer. Moreover, a meta-analysis published in 2020 that included 15 studies compared the diagnostic performance of urinary survivin mRNA with conventional urine cytology. The results demonstrated that survivin mRNA detection achieved 86% sensitivity, 95% specificity, and an AUC value of 0.95—significantly outperforming urine cytology (sensitivity of 42%, AUC of 0.86), particularly in the detection of low-grade tumors. These findings underscore the clinical relevance of survivin as a non-invasive biomarker in detecting bladder cancer (55).

### 4.8 UCA1

UCA1 promotes the initiation and progression of bladder cancer by interacting with BRG1, thereby repressing the expression of p21 and enhancing cell proliferation. At the same time, it activates the PI3K–AKT signaling pathway to inhibit apoptosis and regulates cell-cycle-related genes, resulting in S-phase arrest (56). Furthermore, the long non-coding RNA (lncRNA) UCA1 facilitates bladder cancer progression by recruiting the transcription factor TWIST1 to the promoter regions of IMPDH1 and IMPDH2, thereby upregulating their expression. IMPDH1 and IMPDH2 are rate-limiting enzymes in the *de novo* synthesis of guanine nucleotides. Their upregulation increases guanine nucleotide production, which subsequently stimulates RNA polymerase-dependent preribosomal RNA synthesis and enhances GTPase activity. Collectively, these metabolic effects promote bladder cancer cell growth, motility, and tissue infiltration (57). In a meta-analysis published by Ding et al. in 2021, which included seven studies, it was reported that urinary lncRNA UCA1 achieved a diagnostic sensitivity of 83%, specificity of 86%, and an AUC value of 0.86 in bladder cancer. These findings suggest that UCA1 represents a non-invasive biomarker with high diagnostic accuracy and potential value in clinical auxiliary diagnosis (58).

### 4.9 NMP22

NMP22 is a nuclear matrix protein localized at the mitotic spindle, where it facilitates genome fragmentation and distribution into newly formed nuclei during cell replication. In bladder cancer cells, NMP22 expression is markedly upregulated and released into the urine during apoptosis, making it a potential urinary biomarker for bladder cancer. In addition, NMP22 binds to specific scaffold/matrix attachment regions (S/MARs) within DNA to regulate chromosomal replication and gene transcription. The aberrant expression of NMP22 can alter nuclear architecture and dysregulate critical gene expression, thereby contributing to bladder cancer initiation and progression (59). A comprehensive meta-analysis published by Wang et al. in 2017, encompassing 19 studies with a combined cohort of 5,291 individuals, demonstrated that the urinary NMP22 BladderChek test achieved 56% sensitivity, 88% specificity, and an AUC value of 0.83. These results suggest that NMP22 is suitable for non-invasive detection and recurrence monitoring of bladder cancer, with particularly improved performance observed in Asian populations (60).

### 4.10 Advantages and constraints

This study is the first to apply a Bayesian-based network modeling strategy to systematically integrate and compare multiple non-invasive diagnostic tools for bladder cancer, encompassing two types of sample sources: urine and blood. A total of 26 original studies were included, evaluating the diagnostic performance of seven urinary biomarkers and five blood-based biomarkers, thereby providing a comprehensive assessment of performance differences across biomarker types and sources. Subgroup analyses were further conducted for microRNA biomarkers and combined detection strategies, elucidating their diagnostic performance across different sample sources and offering theoretical support to optimize clinical biomarker combinations. Unlike conventional pairwise comparisons, network meta-analysis synthesizes evidence from both direct comparisons and indirect analyses, enabling the ranking of diagnostic efficacy across multiple methods even in the absence of direct comparative studies. The Bayesian modeling framework, well suited for small sample sizes and complex network structures, demonstrated satisfactory convergence in this study, as indicated by Rhat values consistently close to 1 across all models, reflecting stable and reliable estimates. Moreover, each eligible study underwent methodological assessment using the QUADAS-2 tool, and the analysis was conducted in strict accordance with PRISMA-NMA guidelines to ensure scientific rigor, transparency, and clinical relevance.

Despite the systematic and innovative design of this study, several limitations should be acknowledged. First, although 26 original studies were included to evaluate various non-invasive biomarkers, some markers were supported by only a limited number of studies with small sample sizes, resulting in greater uncertainty in their diagnostic estimates. Second, significant heterogeneity was observed in detection methods, cutoff thresholds, and study designs, which may have affected the consistency of the network. Although Bayesian modeling can partially account for this variability, the risk of bias cannot be fully eliminated. In addition, most primary studies lacked detailed reporting on key oncological parameters such as tumor stage and grade, thereby limiting further stratified analyses of diagnostic performance (e.g., low-grade vs. high-grade, muscle-invasive vs. non-muscle-invasive disease). Another noteworthy issue is that most included studies did not apply correction for multiple testing when evaluating several biomarkers simultaneously. The absence of this statistical adjustment may increase the risk of type I errors, thereby leading to an overestimation of statistical significance in some results. Although a few studies reported using methods such as Bonferroni correction (14, 16, 17, 19, 25, 36), this problem remains common overall. Tissue-derived biomarkers were also excluded from the analysis due to the invasive nature of their sampling procedures (e.g., biopsy or surgery), which are inconsistent with the definition of non-invasive diagnostic tools and limit their applicability in early screening and routine surveillance settings. To address these limitations, future studies should prioritize standardizing detection methods and cutoff values to reduce variability, conducting large-scale multicenter investigations to improve robustness, and performing stratified analyses by tumor stage, grade, and other clinical features to achieve more precise evaluations. In addition, greater attention should be given to applying appropriate multiple testing correction methods when evaluating several biomarkers simultaneously so as to reduce the risk of false positives and improve the reliability of findings. Finally, integrative multi-omics approaches should be advanced to enhance the accuracy and clinical applicability of non-invasive diagnostic tools.

## 5 Conclusions

In conclusion, this study used a Bayesian network meta-analysis to compare the diagnostic performance of non-invasive biomarkers for bladder cancer. This study demonstrated that certain urinary and blood microRNAs, as well as multi-gene combination strategies, have strong potential to support the early detection and follow-up of bladder cancer, serving as an important complement to cystoscopy. Notably, a previous systematic review comprehensively summarized the applications of classical biomarkers such as CYFRA 21.1, ERCC1, p53, FGFR3, and TATI in bladder cancer diagnosis and prognosis and confirmed their significant advantages and clinical potential in early diagnosis, recurrence monitoring, and individualized treatment (61).

Advances in multi-omics, artificial intelligence, and liquid biopsy are driving non-invasive diagnostics toward greater precision and personalization. A recent review highlighted the potential of urine-based liquid biopsy markers—including DNA methylation, exosomal RNAs, proteins, metabolomic signatures, and multi-gene panels—in diagnosis and prognosis, emphasizing the clear advantages of multi-marker and multi-omics strategies in improving diagnostic accuracy (62).

Moreover, artificial intelligence (AI) and machine learning can enhance diagnostic performance through the analysis of large-scale biomarker datasets and support individualized treatment strategies—for example, a support vector machine model (SVM) based on urinary cfDNA fragments demonstrated high diagnostic accuracy in bladder cancer, with an overall sensitivity of 87%, 71% for early-stage lesions, and up to 92% for advanced cases, achieving an AUC value of 0.96 (63). These findings underscore the great potential of AI in non-invasive diagnostics while also highlighting the importance of standardization and clinical translation to ensure reproducibility and reliability.

## Data Availability

The original contributions presented in the study are included in the article/**Supplementary Material**. Further inquiries can be directed to the corresponding authors.

## Supplementary material

Supplementary Table 1 Literature search strategy.
