# Supplementary material for: Integrated assessment of non-invasive diagnostic tools for bladder cancer: a network meta-analysis
Source: Front Oncol. 2025 Oct 17;15:1649420. doi: 10.3389/fonc.2025.1649420 (PMC12575194; doi:10.3389/fonc.2025.1649420)

**TableS1 Literature search strategy**

pubmed：5567

((((((((("Cell-Free Nucleic Acids"[Mesh]) OR (Cell Free Nucleic Acids[Title/Abstract] OR Circulating Nucleic Acid[Title/Abstract] OR Circulating Cell Free Nucleic Acid[Title/Abstract] OR Cell Free Nucleic Acid[Title/Abstract] OR Circulating Cell Free Nucleic Acids[Title/Abstract] OR Circulating Nucleic Acids[Title/Abstract] OR Cell Free DNA[Title/Abstract] OR Cell Free Deoxyribonucleic Acid[Title/Abstract] OR Circulating DNA[Title/Abstract] OR cfDNA[Title/Abstract] OR cirDNA[Title/Abstract])) OR (("Survivin"[Mesh]) OR (Baculoviral IAP Repeat containing Protein 5[Title/Abstract] OR BIRC5 Protein[Title/Abstract] OR AIP4 protein[Title/Abstract] OR apoptosis inhibitor 4[Title/Abstract] OR inhibitor of apoptosis 4 protein[Title/Abstract] OR inhibitor of apoptosis protein 4[Title/Abstract] OR protein AIP4[Title/Abstract] OR protein BIRC5[Title/Abstract] OR survivin[Title/Abstract]))) OR (("MicroRNAs"[Mesh]) OR (micro RNA[Title/Abstract] OR microRNAs[Title/Abstract] OR miRNA[Title/Abstract] OR miRNAs[Title/Abstract] OR Micro RNA[Title/Abstract] OR Small Temporal RNA[Title/Abstract] OR stRNA[Title/Abstract] OR Primary MicroRNA[Title/Abstract] OR pri miRNA[Title/Abstract] OR Primary miRNA[Title/Abstract] OR pre miRNA[Title/Abstract]))) OR (("angiogenin" [Supplementary Concept]) OR (Angiogenin[Title/Abstract] OR angiogenin-1[Title/Abstract] OR ribonuclease 5[Title/Abstract] OR RNase-5[Title/Abstract]))) OR (("Cytology"[Mesh]) OR (Cytopathology[Title/Abstract] OR Cytopathologies[Title/Abstract] OR automated cytological technique[Title/Abstract] OR cell biology[Title/Abstract] OR cytological techniques[Title/Abstract] OR cytotest[Title/Abstract] OR cytology[Title/Abstract]))) OR ((NMP22[Title/Abstract] OR NMP22 protein[Title/Abstract] OR protein NMP22[Title/Abstract] OR nuclear matrix protein 22[Title/Abstract]) OR ("nuclear matrix protein 22" [Supplementary Concept]))) OR (("Fibronectins"[Mesh]) OR (alpha2 opsonin[Title/Abstract] OR beta cryoglobulin[Title/Abstract] OR cell adhesion factor[Title/Abstract] OR cold insoluble globulin*[Title/Abstract] OR large external transformation sensitive protein[Title/Abstract] OR lets glycoprotein*[Title/Abstract] OR lets protein[Title/Abstract] OR fibronectin*[Title/Abstract] OR Opsonic alpha 2 SB Glycoprotein[Title/Abstract] OR alpha 2 Surface Binding Glycoprotein[Title/Abstract] OR Opsonic Glycoprotein[Title/Abstract] OR Cold Insoluble Globulins[Title/Abstract]))) OR (("UCA1 RNA, human" [Supplementary Concept]) OR (UCA1[Title/Abstract] OR urothelial carcinoma-associated 1[Title/Abstract]))) AND (("Urinary Bladder Neoplasms"[Mesh]) OR (Urinary Bladder Neoplasm[Title/Abstract] OR Bladder Neoplasm*[Title/Abstract] OR Bladder Tumor*[Title/Abstract] OR Urinary Bladder Cancer[Title/Abstract] OR Bladder Cancer*[Title/Abstract] OR Cancer of Bladder[Title/Abstract] OR Cancer of the Bladder[Title/Abstract] OR Malignant Tumor of Urinary Bladder[Title/Abstract] OR bladder malignancy*[Title/Abstract] OR Ca bladder[Title/Abstract] OR cancer of the urinary bladder[Title/Abstract] OR carcinomatous bladder[Title/Abstract] OR malignancies of the bladder[Title/Abstract] OR malignancy of the bladder[Title/Abstract] OR malignant bladder neoplasm*[Title/Abstract] OR malignant bladder tumor*[Title/Abstract] OR malignant bladder tumour*[Title/Abstract] OR malignant neoplasm of the bladder[Title/Abstract] OR malignant neoplasms of the bladder[Title/Abstract] OR malignant neoplasms of the urinary bladder[Title/Abstract] OR malignant tumor of the bladder[Title/Abstract] OR malignant tumor of the urinary bladder[Title/Abstract] OR malignant tumors of the bladder[Title/Abstract] OR malignant tumors of the urinary bladder[Title/Abstract] OR malignant tumour of the bladder[Title/Abstract] OR malignant tumours of the bladder[Title/Abstract] OR malignant tumours of the urinary bladder[Title/Abstract] OR malignant urinary bladder tumor[Title/Abstract] OR malignant urinary bladder tumors[Title/Abstract] OR urine bladder cancer[Title/Abstract] OR vesical cancer[Title/Abstract]))

**embase** 15218


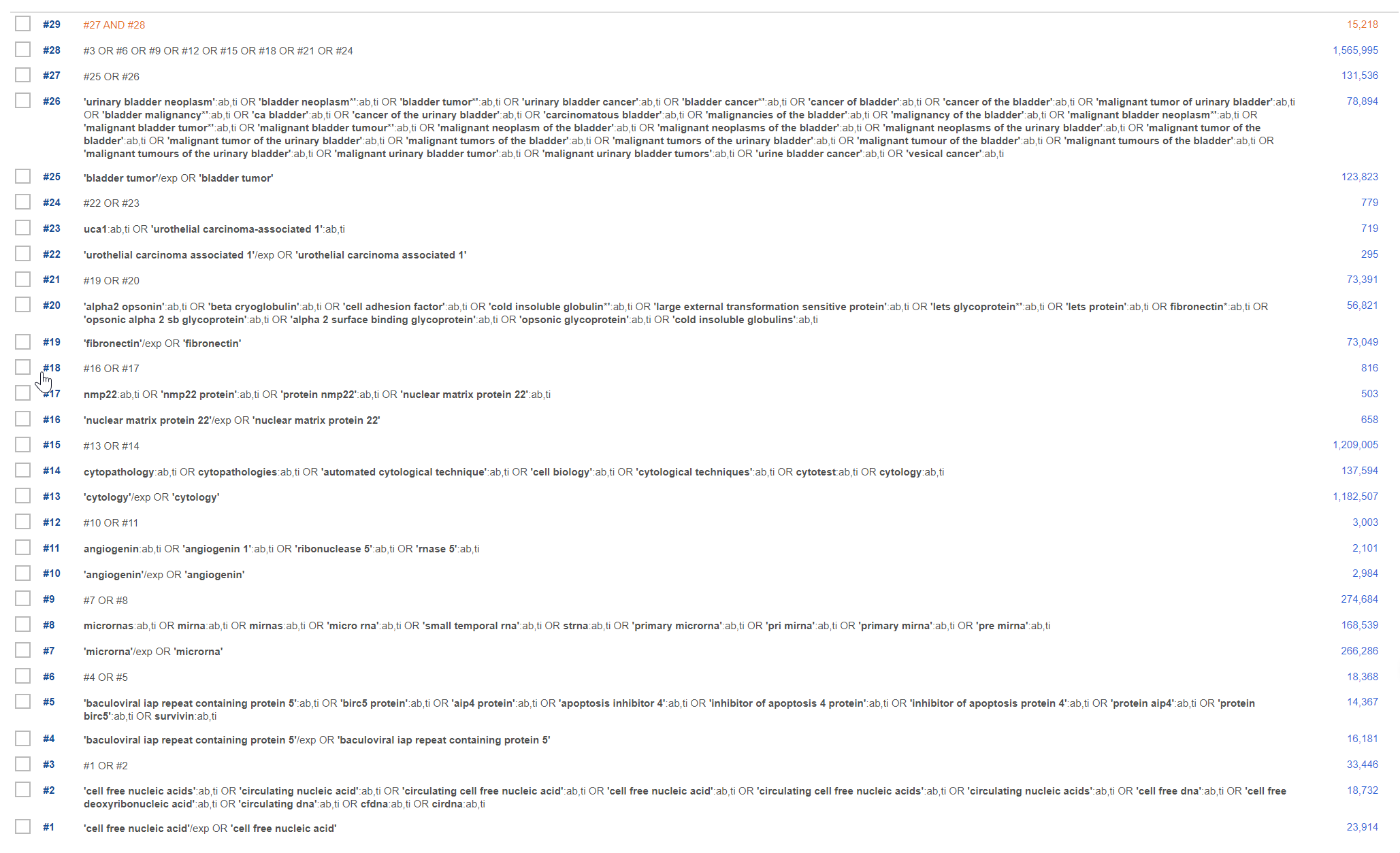


**Cochrance** 601，下载trail 600


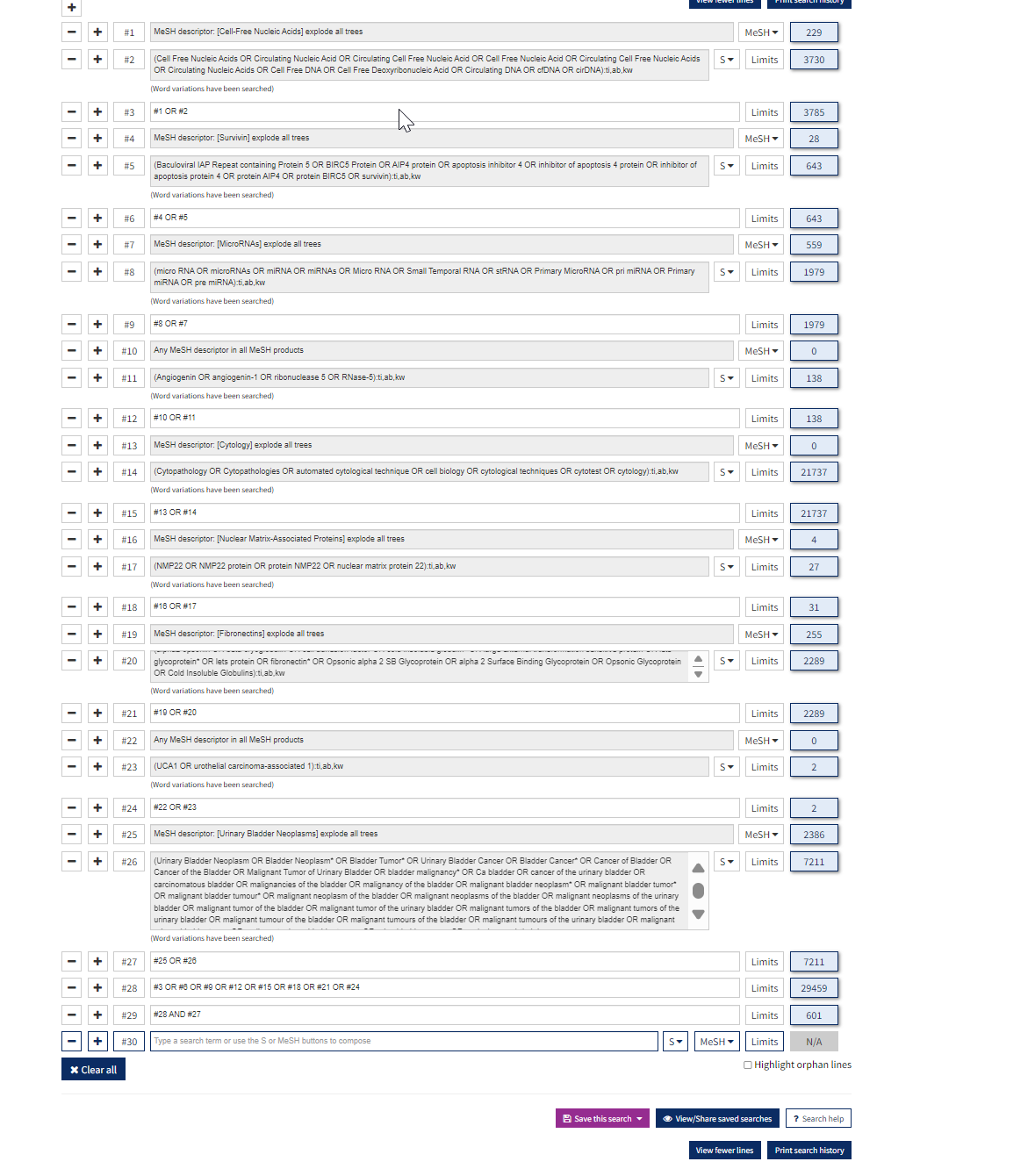


**Web of Science** 8445


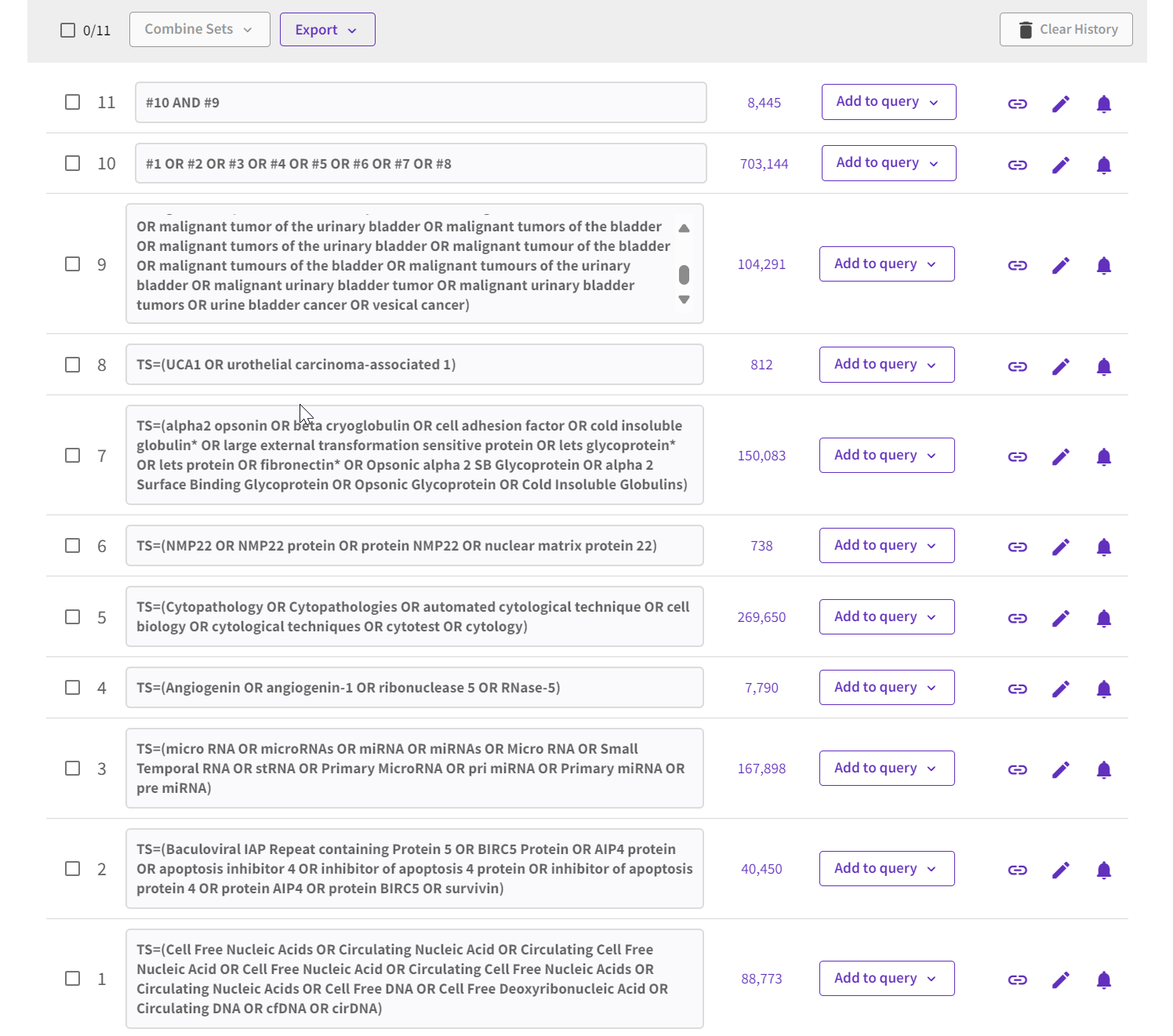

Supplement: Supplementary Table 1 — Literature search strategy. [file Table1.docx]
